# A micropeptide JunBP regulated by TGF-β promotes hepatocellular carcinoma metastasis

**DOI:** 10.1038/s41388-022-02518-0

**Published:** 2022-11-15

**Authors:** Hongwei Zhang, Zhibin Liao, Weijian Wang, Yachong Liu, He Zhu, Huifang Liang, Bixiang Zhang, Xiaoping Chen

**Affiliations:** 1 Hepatic Surgery Center, Tongji Hospital, Tongji Medical College, Huazhong University of Science and Technology, Wuhan, Hubei P.R. China; 2 Hubei Key Laboratory of Hepato-Pancreato-Biliary Diseases, Wuhan, Hubei P.R. China; 3 Key Laboratory of Organ Transplantation, Ministry of Education and Ministry of Health, Wuhan, Hubei P.R. China

**Keywords:** Liver cancer, Prognostic markers

## Abstract

Transforming growth factor beta (TGF-β) signaling pathway plays important roles in hepatocellular carcinoma (HCC) progression. Long intergenic non-protein coding RNAs (lincRNAs) are important components of TGF-β signaling pathway and perform their functions through different mechanisms. Here, we found that LINC02551 was activated by TGF-β transcriptionally and identified a 174-amino-acid peptide, Jun binding micropeptide (JunBP), encoded by LINC02551 in HCC tissues and HCC cell lines. Functional study showed that JunBP promotes HCC metastasis through binding to c-Jun and subsequent promotion of its phosphorylated activation. Activated c-Jun has higher binding affinity to SMAD3, which in turn leads to more SMAD3 recruited to the promoter region of LINC02551. We find a positive feedback among them, and this mechanism provides a novel potential prognostic biomarker and therapeutic target in HCC.

## Introduction

Hepatocellular carcinoma (HCC) is one of the most common malignancies and the third leading cause of cancer-related deaths worldwide [1]. The high mortality rate results from late presentation at advanced stage, high incidence of tumor metastasis, and tumor recurrence after surgical resection [2–4]. Although significant progress has been achieved over the past decades, the outcomes of patients with late-stage HCC are still unsatisfactory [5]. Therefore, the molecular mechanisms of HCC pathogenesis and metastasis remain to be defined.

Transforming growth factor β (TGF-β) is a multifunctional cytokine vitally implicated in the pathogenesis of chronic liver diseases and cancers [6–9]. A number of signaling pathways can be activated by TGF-β in a SMAD-independent manner through direct phosphorylation of downstream effectors, including the Ras/mitogen-activated protein kinase (MAPK), p38, c-Jun N-terminal kinase (JNK) and phosphoinositide 3 kinase (PI3K)/Akt pathways [10–13]. Previous studies showed that SMAD complex had crosstalk with many mediate molecules in these non-canonical pathways [14, 15]. And in our previous studies, we found the critical role of transcriptional intermediary factor 1 gamma (TIF1γ) in the TGF-β/SMAD signaling pathway [16]. We also investigated the roles of canonical TGF-β/SMAD3 signaling and identified one of the downstream effectors, PTPRε, on HCC migration and metastasis [17].

It is found that long intergenic non-protein coding RNAs (lincRNAs) are emerging as a fundamental aspect of biology due to their ability to reprogram gene expression and influence distinct cellular functions. Also, many lincRNAs affect hallmarks of cancers and it has been proved that some lincRNAs can serve as oncogenes or tumor suppressors [18–20]. Mechanistically, lincRNAs have effects on the function of transcriptional complexes [21, 22], the modulation of chromatin structures [23, 24], the formation of ribonucleoprotein (RNP) complexes [25]; furthermore, some lincRNAs can serve as decoys for proteins and micro-RNAs (miRNAs) [26]. In addition, ncRNAs which were considered non-coding might, as a matter of fact, be able to encode small peptides [27, 28]. Previously, these small peptides were filtered out by default and treated as noises or by-product. Nowadays, it is recognized that ncRNAs contain short open reading frames (sORFs) that can be translated into functional small peptides. LincRNAs are described as important components of the TGF-β signaling pathway. These lincRNAs activated by TGF-β promote the invasion-metastasis cascade in different types of cancers [29, 30]. However, the exact roles and mechanisms of TGF-β mediated lincRNAs in HCC remain not fully understood and need to be further investigated.

In this study, we found a previously uncharacterized lincRNA-LINC02551. It encodes a micropeptide which was overexpressed in HCC cell lines upon TGF-β stimulation. It also promotes HCC metastasis partly through binding with c-Jun. Based on its function that it could bind to the bZIP domain of c-Jun, we named it Jun binding micropeptide (JunBP). Finally, we illuminated a possible feedback among SMAD3, JunBP, and c-Jun.

## Results

### LINC02551 is upregulated by TGF-β stimulation

**Fig. 1:**
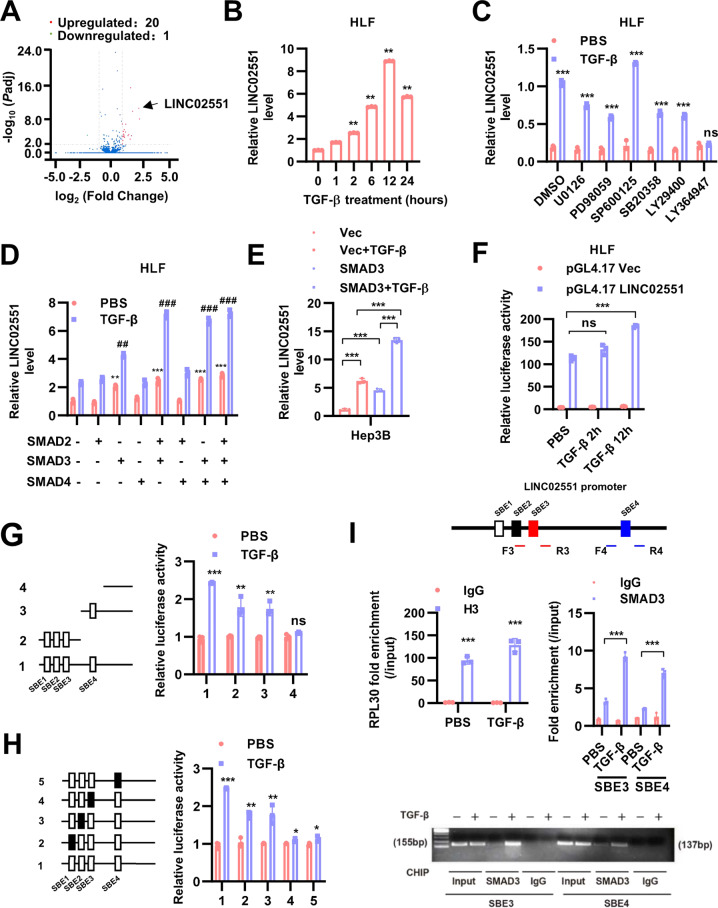
LINC02551 is upregulated upon TGF-β stimulation. **A** Volcano plot of the analysis of the RNA-seq of HLF treated without and with TGF-β. Red plots mean the upregulated genes; green plot means the downregulated genes. **B** qRT-PCR analysis of LINC02551 expression treated with TGF-β for in the HLF cell line. **C** qRT-PCR analysis of LINC02551 levels in HLF cells treated with TGF-β together with different inhibitors in TGF-β signaling. **D** qRT-PCR analysis of LINC02551 expression level in HLF cells transfected with SMAD2/3/4 alone or together. **E** qRT-PCR results of LINC02551 levels in Hep3B-SMAD3 stable cells treated with TGF-β. **F** Luciferase activities of LINC02551 promoter in HLF cells treated with TGF-β. **G** The activity of truncated mutants of LINC02551 promoter in HLF cells. **H** The activity of SBE mutants of LINC02551 promoter in HLF cells. **I** Anti-SMAD3 chromatin immunoprecipitation assays followed by quantitative real-time PCR and reverse transcription PCR analyses was conducted in HLF cells. Anti-H3 is used as a positive control antibody and PRL30 is used as a positive control gene. (mean ± SD, *P < 0.05; **P < 0.01, ***P < 0.001 and ns, *P* > 0.05).

This study was initiated in an attempt to verify lincRNAs which were regulated by TGF-β. Thus, we applied RNA-sequencing (RNA-seq) in HLF cells stimulated with TGF-β (5 ng/ml for 12 h) (Fig. 1A and Supplementary Table S1). Five lincRNAs significantly upregulated upon TGF-β were verify through qRT-PCR in HLF and Hep3B cell lines. LINC02551 appeared to be the top hit due to its concurrent regulation by time-dependent TGF-β stimulation in the two cell lines (Fig. 1B and Supplementary Fig. S1C). And it was also upregulated upon TGF-β stimulation in a dose-dependent manner. (Supplementary Fig. S1D). Compared with LINC02551, the other four lincRNAs did not have accordant responses upon TGF-β stimulation in the two cell lines (Supplementary Fig. S1A, B). To explore how LINC02551 was upregulated by TGF-β, we used different inhibitors in TGF-β signaling pathway to stimulate HLF and Hep3B and found that its upregulation upon TGF-β stimulation was totally abrogated by TGFBR1 inhibitor LY364947 but not other inhibitors (Fig. 1C and Supplementary Fig. S1E). To further confirm the role of SMAD proteins, we tested the effects of ectopic expression of SMAD2/3/4 alone or together and found that SMAD3 overexpression significantly increased the level of LINC02551 upon TGF-β stimulation and the level was maximal when SMAD2/3/4 were all overexpressed (Fig. 1D and Supplementary Fig. S1F). Furthermore, LINC02551 expression was upregulated upon TGF-β stimulation in SMAD3-overexpressed Hep3B (Fig. 1E), while the upregulation was interrupted in SMAD3-knockdown LM3 (Supplementary Fig. S1G).

SMAD3 is reported to be a canonical transcriptional factor together with SMAD2 and SMAD4. To explore whether the regulation was occurred in the transcriptional level, we used the reporter assay and found that LINC02551 is transcriptionally upregulated upon TGF-β stimulation (Fig. 1F and Supplementary Fig. S1H). According to the JASPAR analysis (http://jaspar.genereg.net/), LINC02551 promoter contained 4 putative SMAD3/4-binding elements (SBE) on the minus or plus strand at positions -936, -902, -879 and -639, respectively. After constructing different point and truncated mutants, the reporter assay indicated that SBE-3 and SBE-4 might be responsible for the upregulation of LINC02551 induced by TGF-β stimulation (Fig. 1G, H). Chromatin immunoprecipitation (ChIP) followed by qRT-PCR analyses showed that SMAD3 bound to SBE-3 and SBE-4 regions (Fig. 1I).

These data suggested that SMAD3 might promote the transcription of LINC02551 upon TGF-β stimulation.

### LINC02551 encodes a micropeptide, which is naturally, endogenously produced

**Fig. 2:**
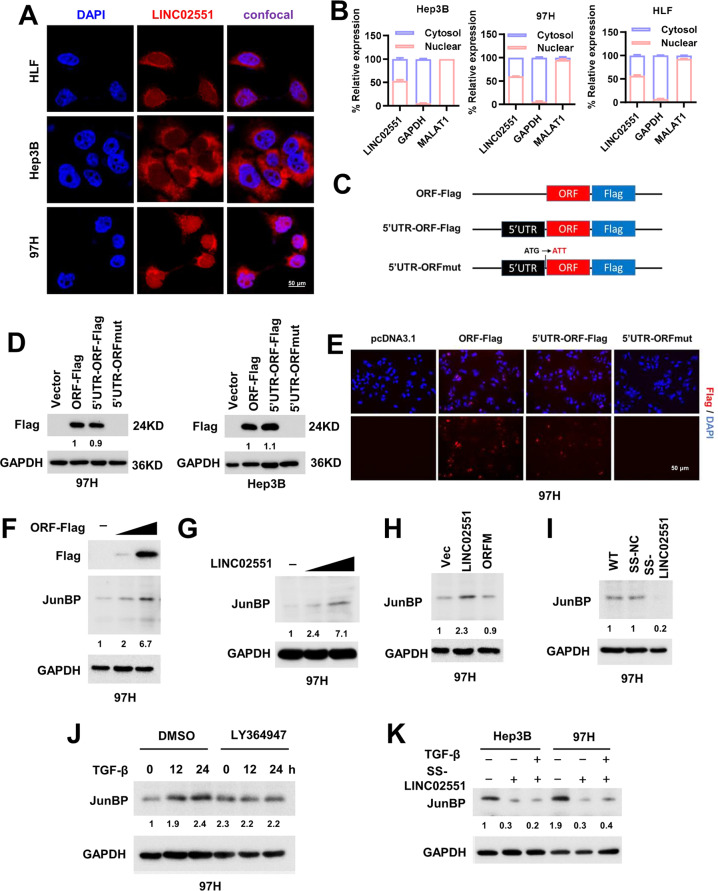
A micropeptide JunBP is naturally and endogenously translated by LINC02551. **A** LINC02551-FISH (fluorescence in situ hybridization) with indicated probes (Cy3 is added to LINC02551) in HCC cells. **B** qRT-PCR analysis of LINC02551 subcellular localization followed by subcellular fractionation (representative from *n* = 3 biological replicates). GAPDH and MALAT1 were used as controls for the cytoplasm and nucleus, respectively. **C** The diagram of the Flag fusion constructs used for transfection. The initiation codon ATG in LINC02551 was mutated to ATT (ORFM). **D** WB results of the three plasmids expression in 97H and Hep3B cells. **E** IF (immunofluorescence) assays in 97H cells. **F** 97H cells were transfected with ORF-Flag in concentration gradients. **G** 97H cells were transfected with LINC02551 in concentration gradients. **H** 97H cells were transfected with Smart Silencer. **I** 97H cells were transfected with LINC02551 or ORFM. **J** The expression of JunBP in 97H cells treated with LY364947 and TGF-β for indicated times. **K** The expression of JunBP in HCC cells transfected with Smart Silencer and stimulated by TGF-β.

To figure out the subcellular localization of LINC02551, we used nuclear/cytoplasm fractionation and confocal microscopy analysis of fluorescent in situ hybridization and found that more than half of LINC02551 was located in the cytoplasm (Fig. 2A, B), suggesting that it might be translated into protein. The rapid-amplification of cDNA ends (RACE) assay confirmed that LINC02551, located on human chromosome 11, was composed of 1455 nucleotides and consisted of six exons (Supplementary Fig. S2A). To determine whether the in-frame ATG initiation codon of LINC02551 was functional, we constructed plasmids by fusing the wild-type (WT) ORFs of LINC02551 and Flag, as well as the mutant versions that ATG initiation codons were mutated to ATT (Fig. 2C). The expression of Flag fusion protein was observed in the 293T cells transfected with ORF-Flag and 5’UTR-ORF-Flag plasmids but not the cells transfected with 5’UTR-ORF-mut plasmid, suggesting that LINC02551 ATG codon was functional (Fig. 2D, E and Supplementary Fig. S2C). Then we got the detailed amino acids sequence of JunBP and after blasting the sequence in NCBI, we found that it was not conservative and only expressed in human and pan paniscus (Supplementary Fig. S2B). To determine whether JunBP was endogenously expressed, we generated a rabbit polyclonal antibody against JunBP (Anti-JunBP). LINC02551, LINC02551-Flag, and ORF-Flag were transfected into 97H cells and all detected by Anti-JunBP (Fig. 2F–H and Supplementary Fig. S2D). When LINC02551 was knocked down by Smart Silencer (RiboBio company), JunBP detected by Anti-JunBP was much lower (Fig. 2H and Supplementary Fig. S2E). These data confirmed the specificity of Anti-JunBP. To further confirm that JunBP was translated from LINC02551 naturally, LY364947 and LINC02551 Smart Silencer were used and we found that the upregulation of JunBP upon TGF-β stimulation was blocked with the inhibition of either TGFBR1 or LINC02551 (Fig. 2J, K and Supplementary Fig. S2G).

Altogether, our results showed that JunBP was naturally and endogenously produced by LICN02551 in HCC cells and was upregulated upon TGF-β stimulation.

### JunBP promoted HCC metastasis in vitro and in vivo

**Fig. 3:**
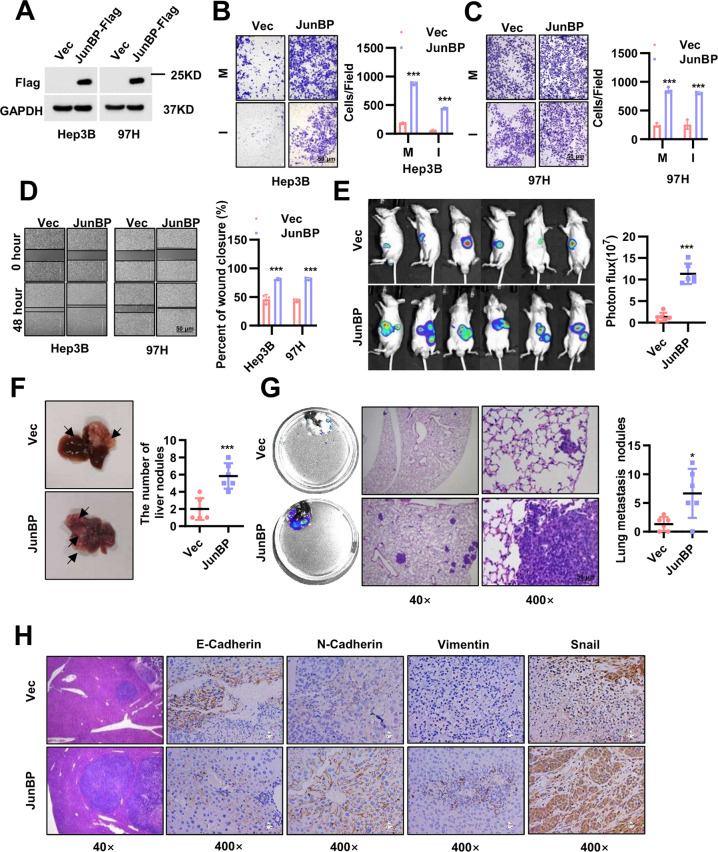
The effect of JunBP overexpression on HCC metastasis. **A** The identification of JunBP-Flag expression in Hep3B and 97H cells. **B** The migration and invasion results of JunBP overexpression in Hep3B cells (M: Migration; I: Invasion). **C** The migration and invasion results of JunBP overexpression in 97H cells. **D** The wound healing assay of JunBP overexpression in Hep3B and 97H cells. **E** Before executed, the mice were injected with luciferase substrate. And the luciferase activities were analyzed in the right graph. **F** The representative images of the mouse liver. And the in situ metastasis nodules were counted. **G** The lungs were separated and perfused by luciferase substrate. The H&E staining of the lung were presented. **H** EMT markers IHC staining in the mouse livers.

To determine the function of JunBP in HCC progression, we checked its expression in different HCC cell lines (Supplementary Fig. S2F). In Hep3B and 97H cells, we overexpressed JunBP stably (Fig. 3A), which led to increased cell migration and invasion and similar effects were observed in wound healing assay (Fig. 3B–D). And also, western blot analysis indicated upregulation of epithelial-mesenchymal transition (EMT) markers in JunBP overexpression cells (Supplementary Fig. S3A). To evaluate the tumor promotive role of JunBP in HCC in vivo, we applied orthotopic xenograft models to examine intrahepatic metastasis of 97H cells after 5 weeks post inoculation (Fig. 3E). Injection of JunBP-overexpressed cells substantially increased the capacity of HCC cells to form secondary lesions in the liver and mice in overexpressed group went so far as to develop lung metastasis in 5 weeks (Fig. 3F, G). Before these mice were executed, their livers were separated and the IHC staining showed strengthened expression of epithelial-mesenchymal transition (EMT) markers in the JunBP-overexpressed group (Fig. 3H and Supplementary Fig. S3B).

In 97H and HLF cells, we knocked down JunBP. And to evaluate the role of JunBP upon TGF-β stimulation, we treated JunBP knockdown cells with TGF--β stimulation. The results showed that the knockdown of JunBP could partially rescue the role of TGF-β (Supplementary Fig. S4A). To further investigate the rescue effects in vivo, we firstly inoculated 97H vector cells or 97H shJunBP cells into the left lobe of livers of nude mice or injected the above two kinds of cells through tail vein in the nude mice. After inoculation, we administered TGF-β (4 μg/kg) once a week for four times. In the fifth week, we separated the mice livers in the orthotopic xenograft groups and examined the fluorescence intensity of the lungs in the tail vein injection groups. H&E staining analyses of the livers and the lungs showed that TGF-β treatment significantly increased the intrahepatic metastasis and the lung metastasis of HCC and knockdown of JunBP could partially rescue the effects of TGF-β (Supplementary Fig. S4B, C).

### JunBP interacted with c-Jun

**Fig. 4:**
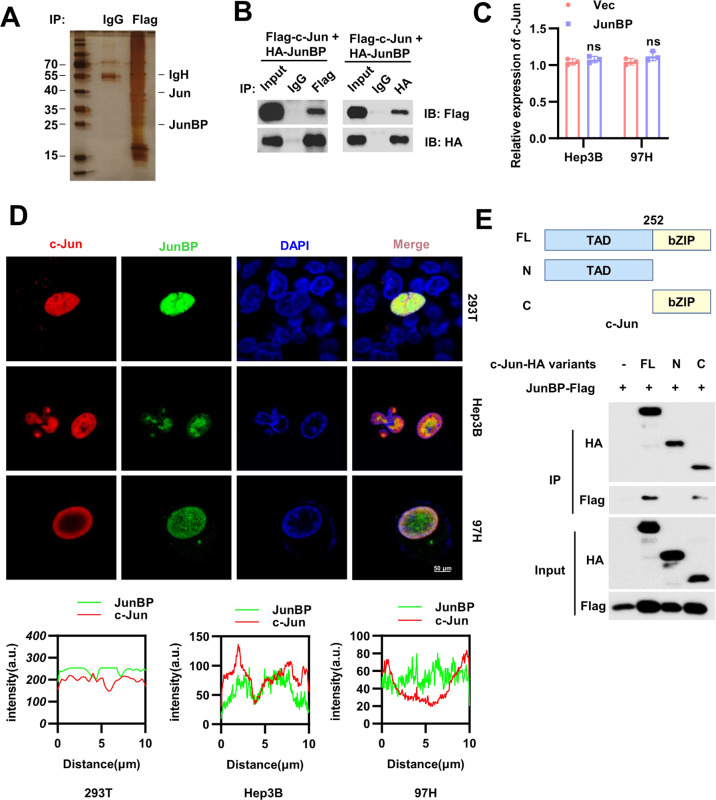
JunBP interacts with c-Jun. **A** CoIP with anti-FLAG, followed by sodium dodecyl sulfate–polyacrylamide gel electrophoresis and silver staining. **B** CoIP analysis between Flag-c-Jun and HA-JunBP in HEK293 cells. **C** qRT-PCR analysis of c-Jun mRNA in Hep3B and 97H cells with JunBP overexpressed. **D** IF results in 293T and HCC cells transfected with Flag-JunBP and HA-c-Jun (the upper panel). The fluorescence intensities in the nucleus were quantified (the lower panel). **E** Upper: the diagram of truncated mutants of c-Jun. Lower: the IP results of JunBP and truncated mutants of c-Jun.

We next explored the underlying mechanism of JunBP-induced promotion of HCC metastasis. Immunoprecipitation (IP) followed by mass spectrometry (MS) assay was used to identify potential JunBP interaction partners (Fig. 4A and Supplementary Table S2). c-Jun was verified to interact with JunBP but did not lead to the change of c-Jun RNA level (Fig. 4B, C). Immunostaining showed that JunBP associated with c-Jun in the nucleus (Fig. 4D). It is reported that c-Jun is composed up of transactivation domain (TAD) and basic-leucine zipper domain (bZIP) [31]. We then constructed two truncated mutants of c-Jun and further analysis showed that JunBP interacted with the bZIP domain of c-Jun (Fig. 4E).

### JunBP promoted the JNK-dependent activation of c-Jun

**Fig. 5:**
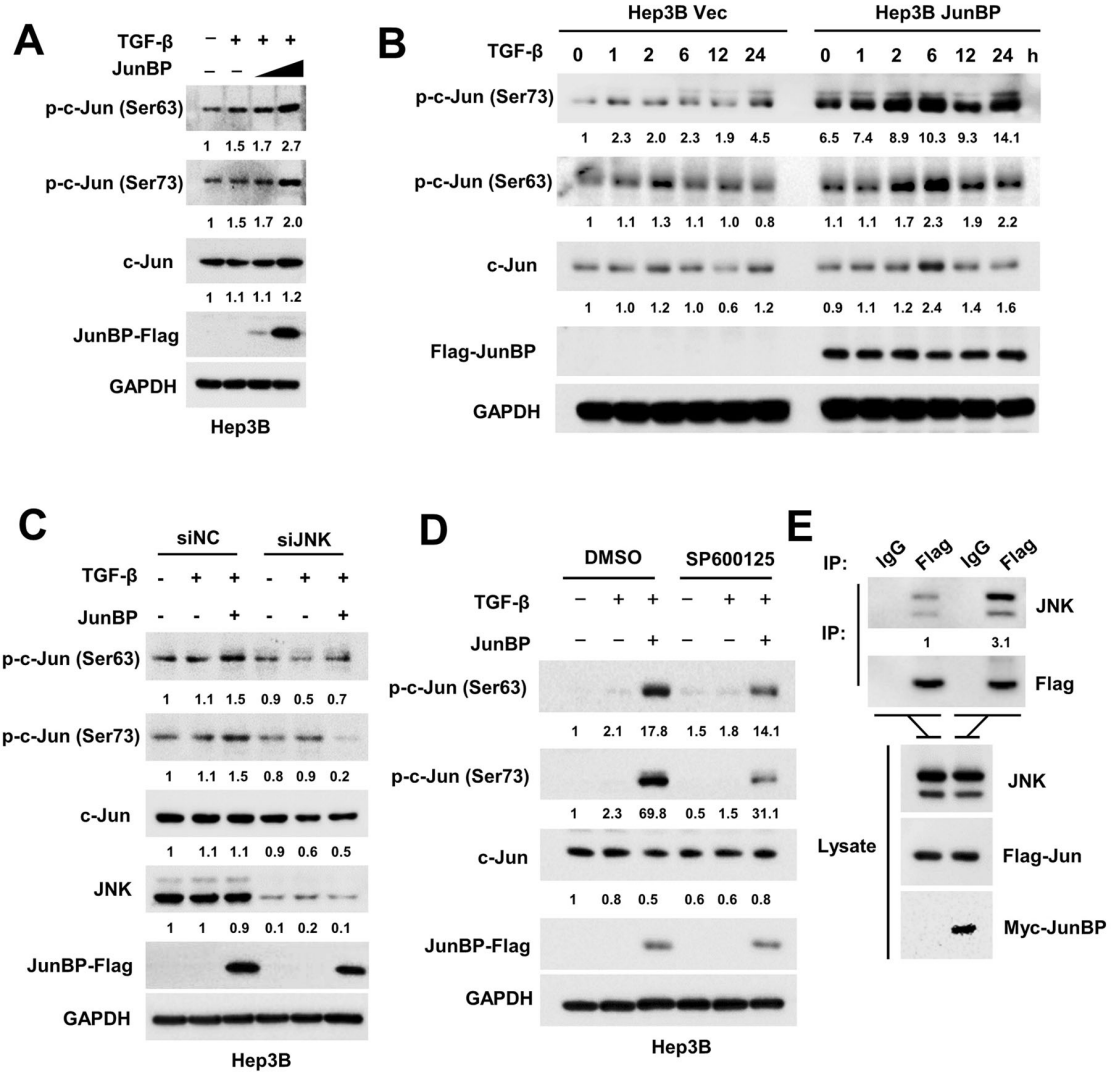
JunBP promotes the JNK-dependent activation of c-Jun upon TGF-β stimulation. **A** The upregulation of phosphorylated c-Jun (p-c-Jun) when transfected with JunBP upon TGF-β stimulation. **B** The upregulation of p-c-Jun in JunBP stably overexpressed Hep3B stimulated with TGF-β in a time-dependent manner. **C** siRNA-mediated knockdown of JNK leads to no change of the expression of p-c-Jun. **D** SP600125 treatment diminished the upregulation of p-c-Jun caused by JunBP. **E** IP analysis between JNK and c-Jun when JunBP was overexpressed.

c-Jun is a vital mediate molecule of mitogen-activated protein kinases (MAPK) signaling pathway [32] and TGF-β could act as one of the extracellular ligands that activate this pathway [33]. When Ser63 and Ser73 of c-Jun are phosphorylated by its canonical upstream kinase JNK upon TGF-β stimulation, it is activated and functions as a transcriptional factor for its many downstream target genes [34]. We next explored whether JunBP has effect on the biological function of c-Jun. JunBP was overexpressed in a dose-dependent manner in HCC cells upon TGF-β stimulation and the results showed that JunBP promoted the activation of c-Jun but not its total protein level (Fig. 5A and Supplementary Fig. S5A). And in JunBP stably overexpressed cell lines, the activation of c-Jun was in line with the TGF-β treatment in a time-dependent manner (Fig. 5B and Supplementary Fig. S5B). When JNK was knocked down (Supplementary Fig. S5C) or its activation was blocked via SP600125 treatment, the upregulation of phosphorylated c-Jun caused by JunBP upon TGF-β stimulation was attenuated (Fig. 5C, D and Supplementary Fig. S5D, E). To investigate whether JNK also interacted with JunBP, endogenous CoIP analyses were conducted in 97H cells and the results indicated that JunBP interacted with c-Jun and JNK in 97H cells (Supplementary Fig. S5F). Further CoIP result showed that JNK had higher binding affinity to c-Jun in the presence of JunBP (Fig. 5E).

Together with these observations, JunBP acts as a bridge for JNK/c-Jun interaction and leads to the more activation of c-Jun upon TGF-β stimulation.

### JunBP promoted the transcription of LINC02551 in a SMAD3-dependent manner

**Fig. 6:**
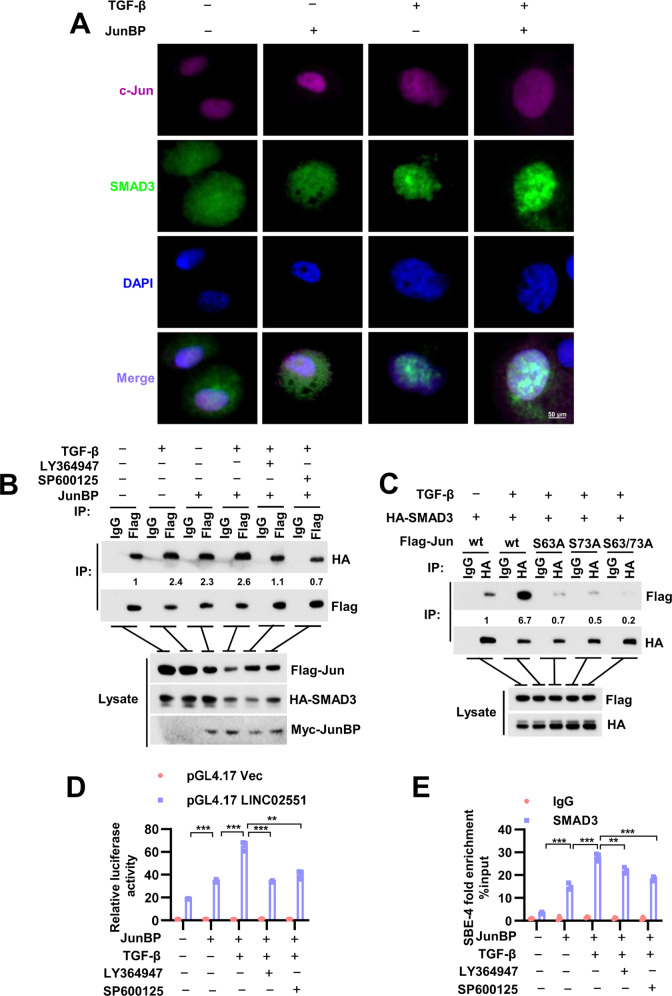
JunBP promotes the activation of SMAD3 and c-Jun upon TGF-β stimulation. **A** IF analysis of the colocalization of SMAD3 and c-Jun when JunBP was overexpressed upon TGF-β stimulation. **B** IP analysis of the combination between SMAD3 and c-Jun when added with LY364947/ SP600125/ TGF-β alone or together. **C** IP analysis of the combination of SMAD3 and different point mutants of c-Jun upon TGF-β stimulation. **D** The luciferase activity of LINC02551 promoter region treated with LY364947 or SP600125 upon TGF-β stimulation. **E** ChIP assays in SBE-4 followed by rt-PCR in 97H cells added with LY364947 or SP600125 upon TGF-β stimulation.

It is reported that SMAD3 can physically interact with AP-1 family members (JunB,c-Jun, and JunD), but the specific modification sites in c-Jun that affect its interaction with SMAD3 remain unstudied [35]. Immunostaining showed that JunBP promoted the interaction between SMAD3 and c-Jun in the nucleus upon TGF-β stimulation (Fig. 6A and Supplementary Fig. S6A). Further IP analysis indicated that the higher binding affinity between SMAD3 and c-Jun caused by JunBP overexpression or TGF-β stimulation was attenuated when the cells used for IP analysis were stimulated by LY364947 (TGFBR1 inhibitor) or SP600125 (JNK inhibitor) (Fig. 6B). To further verify the modification sites in c-Jun that affect its binding affinity with SMAD3, we generated three c-Jun point mutants (S63A, S73A, and S63/73A). The results showed that S63/73A mutant nearly did not interact with SMAD3 upon TGF-β stimulation (Fig. 6C) and additional JunBP expression could not raise the binding affinity between c-Jun and SMAD3 (Supplementary Fig. S6B), which suggested that the activation of c-Jun upon TGF-β stimulation was important for its interaction with SMAD3 and the promotive effect of JunBP was partly dependent on the activation status of c-Jun. AP-1 complex is also reported to act as transcription cofactor for SMAD3 [35]. Based on our previous finding that SMAD3 interacted with the promoter region of LINC02551 to increase its transcription, we did re-ChIP assay using anti-c-Jun antibody and anti-SMAD3 antibody and found that SBE-4 but not SBE-3 was the right binding sequence with c-Jun and SMAD3 in LINC02551 promoter region (Supplementary Fig. S6C).

Above all, we hypothesized that there is a positive feedback among SMAD3, JunBP, and c-Jun upon TGF-β stimulation. We then did reporter assay and found JunBP x’enhanced the luciferase activity of LINC02551; on the contrary, LY364947 or SP600125 treatment led to decreased activity despite the presence of TGF-β stimulation (Fig. 6D). This result was confirmed in the further ChIP assay (Fig. 6E).

These data suggest that JunBP-induced activation of c-Jun in turn leads to the higher binding affinity of SMAD3 to the promoter region of LINC02551 and there is a positive feedback among them.

### JunBP is positively correlated with c-Jun activation in HCC patients

**Fig. 7:**
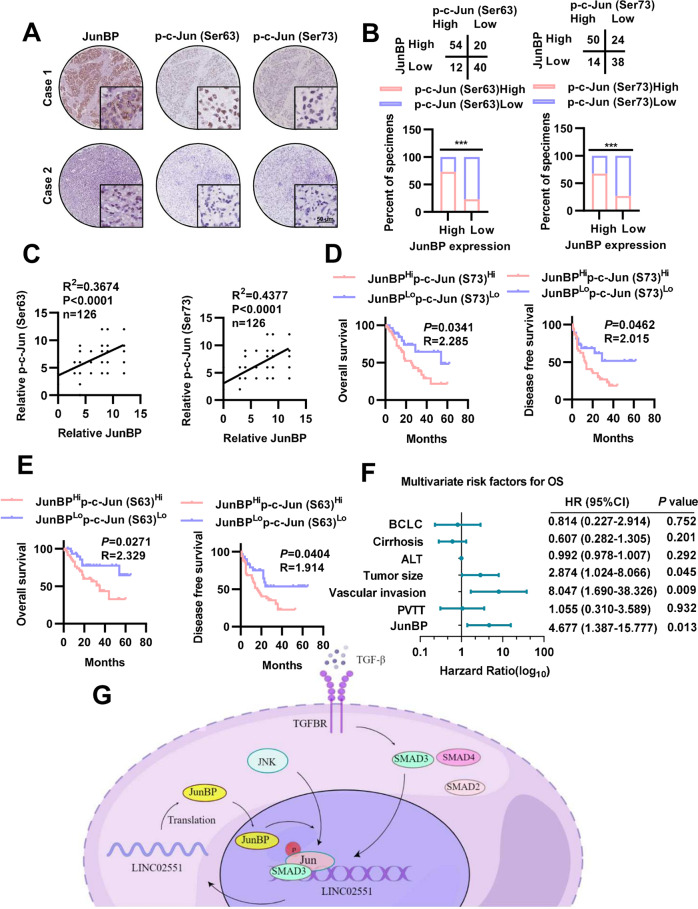
The clinical correlation between JunBP and activated c-Jun. **A** IHC staining of JunBP and activated c-Jun on paraffin-embedded hepatocellular carcinoma tissue from Tongji Hospital. **B** The expression relevance between JunBP and activated c-Jun. **C** The expression relevance of JunBP/p-c-Jun (Ser63) and JunBP/p-c-Jun (Ser73). **D** OS and DFS of patients with high JunBP expression and high p-c-Jun (Ser73) expression. **E** OS and DFS of patients with high JunBP expression and high p-c-Jun (Ser63) expression. **F** Multivariate regression analysis with clinicopathological features significantly associated with prognosis in univariate regression analysis in HCC samples from Tongji hospital. **G** Schematic illustration showing the roles of JunBP in HCC cells.

Immunohistochemistry (IHC) analysis combined with in situ hybridization (ISH) analysis of HCC tissues from Tongji Hospital of Huazhong University of Science and Technology (Wuhan, China) indicated that the expression of JunBP in HCC samples was positively correlated with the expression of the activation status of c-Jun and the expression of LINC02551 (Fig. 7A–C and Supplementary Fig. S7A). After further correlation analyses, the results showed that LINC02551 was positively correlated with SMAD3 and c-Jun (Supplementary Fig. S7A); JunBP was also positively correlated with SMAD3 and c-Jun (Supplementary Fig. S7B, C). Patients with high expression of JunBP had worse overall survival (OS) and disease-free survival (DFS) (Supplementary Fig. S7B). All 126 patients were then divided into different groups based on p-c-Jun (Ser63) and JunBP expression or p-c-Jun (Ser73) and JunBP expression: double-high p-c-Jun (Ser63)/JunBP expression (*n* = 54); double-low p-c-Jun (Ser63)/JunBP expression (*n* = 40); double-high p-c-Jun (Ser73)/JunBP expression (*n* = 50); double-low p-c-Jun (Ser73)/JunBP expression (*n* = 38). After analyzing the prognosis of these patients, we found that HCC patients with high JunBP /high p-c-Jun (Ser63) expression and high JunBP/high p-c-Jun (Ser73) expression had worse DFS and OS (Fig. 7D, E). Combined with the specific information of HCC patients, univariate regression analysis was conducted on the clinicopathological features containing JunBP expression in the cohort of HCC patients from Tongji Hospital, and the results showed BCLC stage, tumor cirrhosis, ALT level, tumor size, vascular invasion, portal vein tumor thrombus (PVTT) and JunBP expression were all correlated with the OS. After multivariate regression analysis of the above factors, we found that JunBP expression was an independent factor associated with OS (Fig. 7F).

## Discussion

In the present study, we identified a micropeptide JunBP encoded by LINC02551, which was transcriptionally upregulated upon TGF-β stimulation. JunBP interacted with c-Jun, increased its JNK-dependent activation, thereby promoted the interaction between SMAD3 and c-Jun, and finally enhanced the transcription of LINC02551 in a positive feedback.

TGF-β signaling induces EMT, invasion, and metastasis in advanced stage of HCC [36]. Previously, we found the suppressive role of TIF1γ in advanced HCC which is dependent on TGF-β stimulation and we also found the prometastatic roles of PTPRε in HCC progression. It has been reported that lincRNAs were activated upon TGF-β stimulation and promoted the invasion of many types of cancers. SMAD2 and SMAD3 are the two major receptor-SMADs (R-SMADs) and SMAD4 is the only cooperative-SMAD (co-SMAD) in TGF-β signaling pathway [37]. R-SMADs are phosphorylated-activated and then combine with SMAD4 upon TGF-β stimulation [38]. The SMAD complexes are thereafter translocated into nucleus to act as transcription factors. To elucidate the mechanism of TGFβ-mediated LINC02551 and JunBP upregulation, we found that SMAD3 overexpression but not SMAD2/4, promoted the level of LINC02551 upon TGF-β stimulation through binding to the promoter region of LINC02551, suggesting that TGF-β upregulates LINC02551 in a SMAD3-dependent manner. When TGFBR1 inhibitor existed or LINC02551 was silenced, the upregulation of JunBP induced by TGF-β decreased, suggesting that TGF-β promotes the translation of JunBP in SMAD3/LINC02551-dependent manner.

Since lincRNAs could encode micropeptides in the cytoplasm, it is necessary to check the coding potential of lincRNAs after verifying their subcellular localization. And the functions of many micropeptides in pathological processes are well studied. Peptide SMIM30 encoded from LINC00998 promoted the HCC tumorigenesis [39]. A micropeptide XBP1SBM encoded by lincRNA MLLT4-AS1 promoted the angiogenesis and metastasis in triple-negative breast cancer (TNBC) [40]. Not all the micropeptides encoded by lincRNA have biological functions. As for lincRNA EPR, it is the RNA itself, but not the encoded peptide, reduces breast cancer cell proliferation [41]. We verified that JunBP was naturally and endogenously expressed in HCC cells and checked its pathological roles. Moreover, we showed that the expression of JunBP was associated with the OS and DFS of HCC patients, which supported the hypothesis that JunBP is a meaningful micropeptide in HCC. But the multivariate regression analysis indicates that JunBP is an independent factor associated with OS but not DFS. Restricted to the number of the HCC patients, further analysis needs to be conducted to verify the specific reasons.

Using mouse model, we showed that JunBP promotes HCC metastasis. This is consistent with other in vitro results: the overexpression of JunBP in 97H and Hep3B increases cell migration and invasion. In situ liver metastasis of mouse intrahepatic transplantation model and the upregulation of some epithelial-mesenchymal transition (EMT) markers were greatly induced by JunBP overexpression, which supported the hypothesis that the dysregulation of EMT markers appear when cells develop stronger motility.

Furthermore, we showed that JunBP was bound to the bZIP domain of c-Jun. Functionally, JunBP promoted the interaction between JNK and c-Jun upon TGF-β stimulation, leading to the activation of c-Jun. Because SMAD3/4 can physically interact with AP-1 family members and associate with an endogenous form of c-Jun that is rapidly phosphorylated in response of TGF-β [35], JunBP also promotes the interaction between SMAD3 and c-Jun which is dependent on the phosphorylation of c-Jun. When their combination is enhanced, we find that SMAD3 has higher affinity to the promoter region of LINC02551, leading to the its transcription. Our data support the hypothesis that there is a positive feedback among JunBP/c-Jun/SMAD3.

It needs to be noted that this study only focuses on the mechanism of JunBP involved in MAPK pathway. However, the biological function of JunBP is complex and broad. In the IP results, JunBP binds to many other proteins, including TP53, YBX1 and CEBPZ and so on. And whether their combinations also have tremendous meanings remains further investigated. LINC02551 is not only located in the cytoplasm, whether the other part located in the nucleus has functions also remains explored.

In summary, we identified a lincRNA-encoding micropeptide JunBP, which was upregulated upon TGF-β stimulation in HCC cells. JunBP was translated from LINC02551, which was transcriptionally upregulated by SMAD3, and JunBP promoted the activation of c-Jun, which in turn promoted the higher binding affinity of SMAD3 to the promoter region of LINC02551 (Fig. 7G). Our study highlights the fact that JunBP, a micropeptide encoded by lincRNA, is a new regulator in HCC and might serve as a potential prognostic biomarker and therapeutic target for HCC.

## Materials and methods

Detailed information is presented in the Supplementary information.

## Supplementary information

Supplementary material
