# Supplementary material for: A micropeptide JunBP regulated by TGF-β promotes hepatocellular carcinoma metastasis
Source: Oncogene. 2022 Nov 15;42(2):113–23. doi: 10.1038/s41388-022-02518-0 (PMC9816058; doi:10.1038/s41388-022-02518-0)
Supplement: Supplementary file 1 — Supplementary material [file 41388_2022_2518_MOESM1_ESM.docx]

**Supplementary Materials and Methods**

**Patient samples**

Human liver tissue samples were collected from patients who underwent surgical resection between 2010 and 2014 at Hepatic Surgery Centre, Tongji Hospital of Huazhong University of Science and Technology (Wuhan, China). These 126 paired tissues were embedded into paraffin. Written informed consent for data analysis was obtained from all patients before operation. Ethical approval was obtained from the Ethic Committee of Tongji Hospital of HUST (TJ-IRB20211214).

**Cell lines and cultures**

Human embryonic kidney cell line HEK293, human normal liver cell line HL7702, and human hepatoma cell line HepG2, HCC cell lines Hep3B, ALEX and HLF were purchased from China Center for Type Culture Collection (CCTCC, Wuhan, China). HCC cells lines MHCC-97H and HCC-LM3 were obtained from Liver Cancer Institute, Zhongshan Hospital, Fudan University, Shanghai, China. All cell lines were cultured in Dulbecco’s modified Eagle’s medium supplemented with 10% fetal bovine serum (Gibco, Grand Island, NY, USA) and maintained at 37 °C in a 5% CO2 incubator.

**Antibodies and reagents**

Mouse serum IgG (I5381 for IP control), rabbit serum IgG (I5006 for IP control), primary antibodies against Flag (F1804 for WB, IP and IF) and HA (H6908 for WB, IP and IF) were from Sigma (St. Louis, MO, USA); primary antibodies against c-Jun (#9165 for WB, IP, IHC-P, IF-IC, CHIP, CHIP-seq and F), phospho-c-Jun-Ser63 (#2361 for WB and IHC), phospho-c-Jun-Ser73 (#3270 for WB, IP, IF-IC, F, IHC-P, CHIP and CHIP-seq) and JNK (#9252 for WB) were from CST (Danvers, MA, USA); and primary antibodies against GAPDH (#60004-1-Ig for WB, IP, IF and ELISA) were purchased from Proteintech (Wuhan, Hubei, China). HRP-conjugated anti-Rabbit IgG (#111-035-003 for WB) and HRP-conjugated anti-Mouse IgG (#115-035-003 for WB) were from Jackson ImmunoResearch Laboratories (PA, USA). HRP-conjugated anti-Rabbit IgG Light Chain (A25022 for WB), HRP-conjugated anti-Mouse IgG Light Chain (A25012 for WB), FITC-conjugated Goat anti-Mouse IgG (A22110 for IF), DyLight549-conjugated Goat anti-Rabbit IgG (A23320 for IF), DyLight549-conjugated Goat anti-Mouse IgG (A23310 for IF) and DyLight649-conjugated Goat anti-Rabbit IgG (A23620 for IF) were purchased from Abbkine (California, USA). All inhibitors were purchased from Selleck (Houston, TX, USA).

**Anti-JunBP antibody preparation**

Peptide synthesis and anti-LINC02551-JunBP antibody preparation were performed by Proteintech (Wuhan, Hubei, China). Briefly, a peptide CDDNHQAQELKRNGANVAE was synthesized, and polyclonal antibodies against the LINC02551-JunBP peptide were obtained from two inoculated rabbits. Antibodies were purified using affinity chromatography on columns containing the corresponding peptides. According to our tests, final purified antibody No. 2 was the correct antibody to recognize the LINC02551-JunBP peptides.

**Plasmids and constructions**

LINC02551 1.0 kb promoter, promoter truncations, SBE site mutations, Jun 1.0 kb promoter sequence and 6×AP-1 sequences were cloned into the pGL4.17 vector. Mammalian expression plasmids for Flag- and HA-tagged JunBP, c-Jun and DDX24 were constructed by standard molecular cloning method from cDNA templates. LINC02551 point mutation plasmids (ORFM with and without Flag-tag) and c-Jun truncated mutation plasmids were constructed by site-directed mutagenesis. All constructs were confirmed by DNA sequencing.

**RNA extraction and qRT-PCR**

Total RNA was isolated from cells and tissues using the RNA Isolater Total RNA Extraction Reagent (Vazyme). First-strand cDNA was synthesized with the Superscript II-reverse transcriptase kit (Vazyme). All qRT-PCR primers are listed in as follows: GAPDH-F: 5’-GACAAGCTTCCCGTTCTCAG-3’ and GAPDH-R: 5’-GAGTCAACGGATTTGGTCGT-3’; LINC02551-F: 5’-GTGCTAGTCTCTGGAGCCAC-3’ and LINC02551-R: 5’-CGGCCACATTTGCACCATTT-3’; CCDC144NL-AS1-F: 5’-CCTGCCAGCTTTGCCTTTAA-3’ and CCDC144NL-AS1-R: 5’-CCGTGTGAGGGAAGTAGTGT-3’; MIAT-F: 5’-TTGCAGGAGAGAGAAGTGGG-3’ and MIAT-R: 5’-ACTGGAGGTGAGGCATGAAA-3’; NKILA-F: 5’-CCCACCTCCAGCCTCTTAAA-3’ and NKILA-R: 5’-AGTCGGTGTCAGGTTGAGTT-3’; AP000695.1-F: 5’-AATCTGGTGTGGACAAACGC-3’ and AP000695.1-R: 5’-AACAGATGCTTCCGAATGCC-3’; c-Jun-F: 5’-AGCAGCAAAGAACTTTCCCG-3’ and c-Jun-R:

5’-CGTCCTTCTTCTCTTGCGTG-3’;

**RNA interference**

LINC02551 Smart Silencer (the target sequences are as follows: 5’-TCACTGAGTTAAACTGCCTA-3’, 5’-GGAAACAAAGCCAAGCGCCG-3’, 5’-GAAAGAAGAAAGACGCACAG-3’, 5’-TGTCAACTCCACCTTTAGA-3’, 5’-GTTCAACGGAAATTCACAA-3’ and 5’-TGCCTTGAATAAAGACGTA-3’) were purchased from Ribo Bio Co., Ltd. (Guangzhou, China). Transfections with Smart Silencer were performed with Lipofectamine 3000 (Life Technologies).

**Transwell cell migration and invasion assay**

Cell migration and invasion assays were performed in a 24-well transwell plate (8 μm pore size, Corning, NY, USA) according to the manufacturer's instruction. For the cell invasion assay, filters were pre-coated with 50 μl 1:4 mixture of Matrigel (BD Biosciences, NJ, USA) and DMEM for 4 hr at room temperature. Culture medium containing 10% FBS was added to lower chambers and aliquots of 5×104 cells in 100 μl of FBS-free medium were seeded into upper chambers. After 24 hr to 48 hr of culturing, non-migrated or non-invaded cells were removed by scraping the membranes with a cotton swab from the upper surface. Cells on the lower surface of the filters were fixed with paraformaldehyde and stained with crystal violet. Cell numbers were counted under an optical microscope. Each experiment was repeated at least three times.

**Wound healing assay**

A confluent monolayer of HCC cells was cultured overnight and a scratch was introduced with a 10 µl pipette tip. Cell migration was recorded by a phase contrast microscopy (Nikon Digital ECLIPSE C1 system, Nikon Corporation) at 0, 24 and 48 hr after the scratch. Photographs of 5 random fields were captured for quantification analysis. Each experiment was repeated at least three times

**Immunoprecipitation**

Co-immunoprecipitation (CoIP) experiments were performed as described previously {Zhang, 2017 #3834}. The specific reagent components were seen in our previous research. Briefly, cells were collected and lysed in IP-lysis buffer. Supernatants were collected by centrifugation (15,000 g, 15 min, 4°C), and were pre-cleared with 30 µl protein G-conjugated agarose (GE Healthcare Life Sciences) followed by centrifugation (2,000 g, 2 min, 4°C). The pre-cleared supernatants were incubated with the indicated antibodies (1 µg/ml) for 3 hr or overnight at 4°C, followed by immunoprecipitation with 20 µl protein G-conjugated agarose for 2 hr at 4°C. The precipitates were washed 5-7 times with IP-wash buffer and detected through WB. For endogenous CoIP experiments, precipitates were washed 3 times with IP-lysis buffer instead, and detected through WB.

**Immunofluorescence**

Cells were fixed with 4% paraformaldehyde for 15 min, followed by permeabilization with 0.5% Triton X-100 for 20 min at room temperature. Primary antibodies (0.2 µg/ml) were added for 2 hr at room temperature post blocking with 5% bovine serum albumin for 1 hr. FITC or DyLight549-conjugated Goat anti-Mouse IgG and DyLight549 or DyLight649-conjugated Goat anti-Rabbit IgG were used as secondary antibodies. Nuclei were counterstained with DAPI. F-actin stress fibers were stained with Alexa Fluor 555-conjugated Phalloidin (Life Technologies). Images were taken by confocal laser-scanning microscopy on a Nikon Digital ECLIPSE C1 system (Nikon Corporation).

**Immunohistochemistry**

Immunohistochemical staining for tissues was performed by using the polymer HRP detection system (Zhongshan Goldenbridge Biotechnology) on paraffin-embedded hepatocellular carcinoma tissue. The paraffin sections were de-waxed and antigen retrieval with 0.01 M sodium citrate buffer (pH 6.0), followed with 3 % hydrogen peroxide incubated for 15 min at room temperature to block endogenous peroxidase, next with 5% bovine serum albumin blocking for 60 min. Primary antibodies were incubated overnight at 4℃ in a humidified chamber, followed by HRP conjugated secondary antibody incubation for 45 minutes at room temperature. Antibody binding was detected by DAB and reaction was stopped by immersion of tissue sections in distilled water once brown color appeared. Tissue sections were counterstained by hematoxylin, dehydrated in graded ethanol. Appropriate positive and negative controls were included for each run of IHC. The immunohistochemical staining and in situ hybridization was scored according to the staining intensity score and percentage of positive stained tumor cells. The rules of the staining intensity scoring were as follows: 0 points (Negative); 1 point (Light brown); 2 points (Brown); 3 points (Dark brown). The rules of stained positive cells scoring were as follows: score 0 denotes less than 10%, score 1 denotes 10-25%, score 2 denotes 26-50%, score 3 denotes 51-75% and score 4 denotes more than 75% of positive stained tumor cells. Overall scores of <6 and ≥6 were defined as negative and positive, respectively.

**Chromatin immunoprecipitation**

Chromatin immunoprecipitation (ChIP) assays were performed using the Chromatin Immunoprecipitation Kit (CST) according to the manufacturer’s protocol. Briefly, Sonication was performed using QSonica Q500 instrument and a microtip probe with 5 sec pulse and 10 sec rest in between for 4-5 cycles with output intensity set at 30 %. Immunoprecipitations were carried out using anti-SMAD3. As for the re-ChIP experiments, we followed the manufacturer’s protocol until part six: Elution of Chromatin. After transferring eluted chromatin supernatant to a new tube, we reconducted part five: chromain immunoprecipitation using anti-Jun. The following procedures are not changed. An isotype-matched IgG was used as the negative control and anti-H3 as the positive control. The immunoprecipitated DNA fragments were detected by real-time PCR assays using appropriate primers. All assays were repeated three times. ChIP primers used for SBE3 and SBE4 regions of LINC02551 1.0 kb promoter were as follows: SBE3-F: 5’-GCTGTTTGTCTGCTGGAATTACC-3’ and SBE3-R: 5’-GCAGGAGAGAGTACAAACCAACA-3’; SBE4-F: 5’-TGTTGGTTTGTACTCTCTCCTGC-3’ and SBE4-R: 5’-GGTGGGAAGAGACAACTGTCAA-3’.

**RNA-Seq**

HLF cells were serum-starved for 12h, followed by TGF-β (5ng/ml for 12h) were lysed with Trizol (Sigma). And then RNA extraction, library preparation, transcriptome sequencing and data analysis were carried out by Novogene Company (Beijing, China).

**Subcellular fractionation**

The subcellular localization of LINC02551 was investigated using PARIS^TM^ Kit according to the manufacturer’s protocol (Thermo Fisher Scientific).

**Rapid amplification of cDNA ends (RACE)**

5’ RACE and 3’ RACE were performed using SMARTer® RACE 5’/3’ Kit according to the manufacturer’s protocols (Takara Bio USA, Inc). In 3’ RACE, cDNAs were generated using an Oligo-dT primer (T20) that complemented the natural polyA tail of mRNAs. PCR was then used to amplify cDNA product from the 3’ end of LINC02551 with a sense specific primer and T20. In 5’ RACE, an antisense gene specific primer was used to produce cDNA from the 5’ end of LINC02551. Next, a string of identical nucleotides (dATP) were added to the 3’ end of the cDNA. PCR was then carried out to amplify cDNA from the 5’ end using an antisense specific primer and T20. We used a priming strategy in which both the 5’ and 3’ RACE reactions were primed using the same primer sequence, albeit reverse complemented, to ensure amplification of a contiguous long transcript.

**Fluorescence in situ hybridization**

LINC02551 FISH probe was synthesized by Ribo Bio Technology Co Ltd (Guangzhou, China). FISH was performed with the FISH kit according to the manufacturer’s protocol (Ribo Bio Tech). Cells were fixed with 4% paraformaldehyde for 10 min at room temperature, and then permeabilized in PBS with 0.5% Triton X-100 on ice for 5 min. Followed by pretreatment with pre-hybridization buffer at 37 °C for 30 min. Subsequently, cells were hybridized with 20 μM using Cy3-labeled RNA of LINC02551 FISH probe mix in a moist chamber at 37 °C overnight. Cells were rinsed thrice in 4 × SSC with 0.1% Tween-20 for 5 min at 42 °C, followed by washing once for 5 min at 42 °C in 2 × SSC and then washed once for 5 min at 42 °C in 1 × SSC. After hybridization, cells were stained with 6-diamidino-2-phenylindole (DAPI) for 10 min at room temperature. Finally, the images were observed with confocal microscope and analyzed with LAS AF Lite (Leica, Solms, Germany).

**Luciferase reporter assay**

Luciferase activity was detected with the Dual-Luciferase Reporter Assay System (Promega, Madison, WI, USA) according to the manufacturer’s instructions. The relative luciferase activity was determined by a GloMax 20/20 Luminometer (Promega). Luciferase activity was normalized to Renilla activity.

**Animal studies**

All animal experiments were performed in accordance with the guidelines for the care and use of laboratory animals and were approved by the Ethic Committee of Tongji Hospital of HUST. The orthotopic models were used to study in vivo metastasis. Briefly, 97H cells (1×10^6^) with or without JunBP stable overexpression were injected into the left lobe of livers of 5-week-old male BALB/C nude mice. The mice were randomly separated with 6 mice in each group. 97H cells with or without JunBP stable knockdown were also inoculated into the livers of 5-week-old male BALB/C nude mice to construct orthotopic xenograft model; 97H cells (1×10^6^) were injected through tail vein in nude mice to construct lung metastasis model. The mice were also randomly separated with 6 mice in each group. Luciferase bioluminescence of each mouse was captured at the fifth week after injection, respectively. All the groups were sacrificed 5 weeks after injection.

**Statistical analysis**

Statistical analyses were performed using SPSS 13.0 (SPSS, Chicago, IL, USA) or GraphPad Prism 5.0 (GraphPad, La Jolla, CA, USA) software. All values are expressed as the mean ± SEM. Paired or unpaired Student’s *t* test, Wilcox test were used for various types of data comparison. Categorical data were analyzed by χ2 test. Kaplan-Meier and log-rank analysis was used to assess the survival between subgroups. The statistical signiﬁcance between data sets was expressed as *P* values, and *P*<0.05 was considered statistically signiﬁcant (*, *P*< 0.05; **, *P*< 0.01; ***, *P*< 0.001).

**Supplementary Figures**


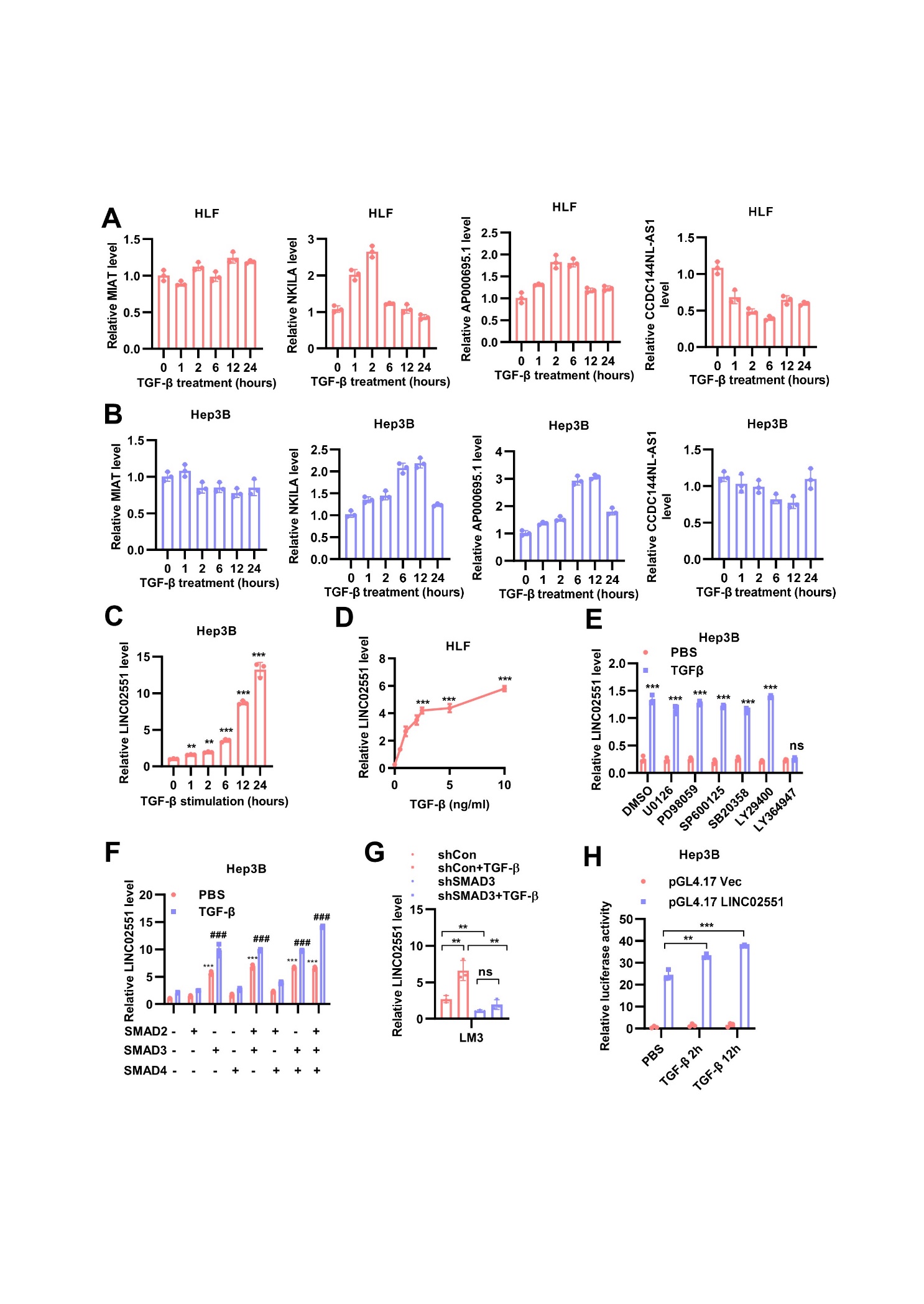


**Figure S1. The regulation of LINC02551 upon TGF-β stimulation. A-B** qRT-PCR analysis of MIAT, NKILA, AP000695.1 and CCDC144NL-AS1 expression in HLF and Hep3B cells, which were serum-starved (2% FBS, 16 h) and then treated with TGF-β (5ng/ml) in a time-dependent manner. **C)** qRT-PCR analysis of LINC02551 expression in Hep3B cells. **D)** qRT-PCR analysis of LINC02551 expression level in HLF with different concentration of TGF-β. **E)** qRT-PCR analysis of LINC02551 levels in Hep3B cells treated with TGF-β together with LY364947 (TGFBR1 inhibitor), LY29400 (AKT inhibitor), U0126 (ERK inhibitor), PD98059 (MEK inhibitor), SP600125 (JNK inhibitor), SB203580 (P38 inhibitor) for 24 h. **F)** qRT-PCR analysis of LINC02551 expression in Hep3B cells transfected with SMAD2/3/4 alone or together upon TGF-β stimulation. **G)** qRT-PCR analysis of LINC02551 levels in LM3-shSMAD3 stable cells upon TGF-β stimulation. **H)** The luciferase activity of LINC02551 promoter in Hep3B cells treated with TGF-β for the indicated times. (mean ± SD, **P* < 0.05; ***P* < 0.01, ****P* < 0.001 and ns, *P* > 0.05).


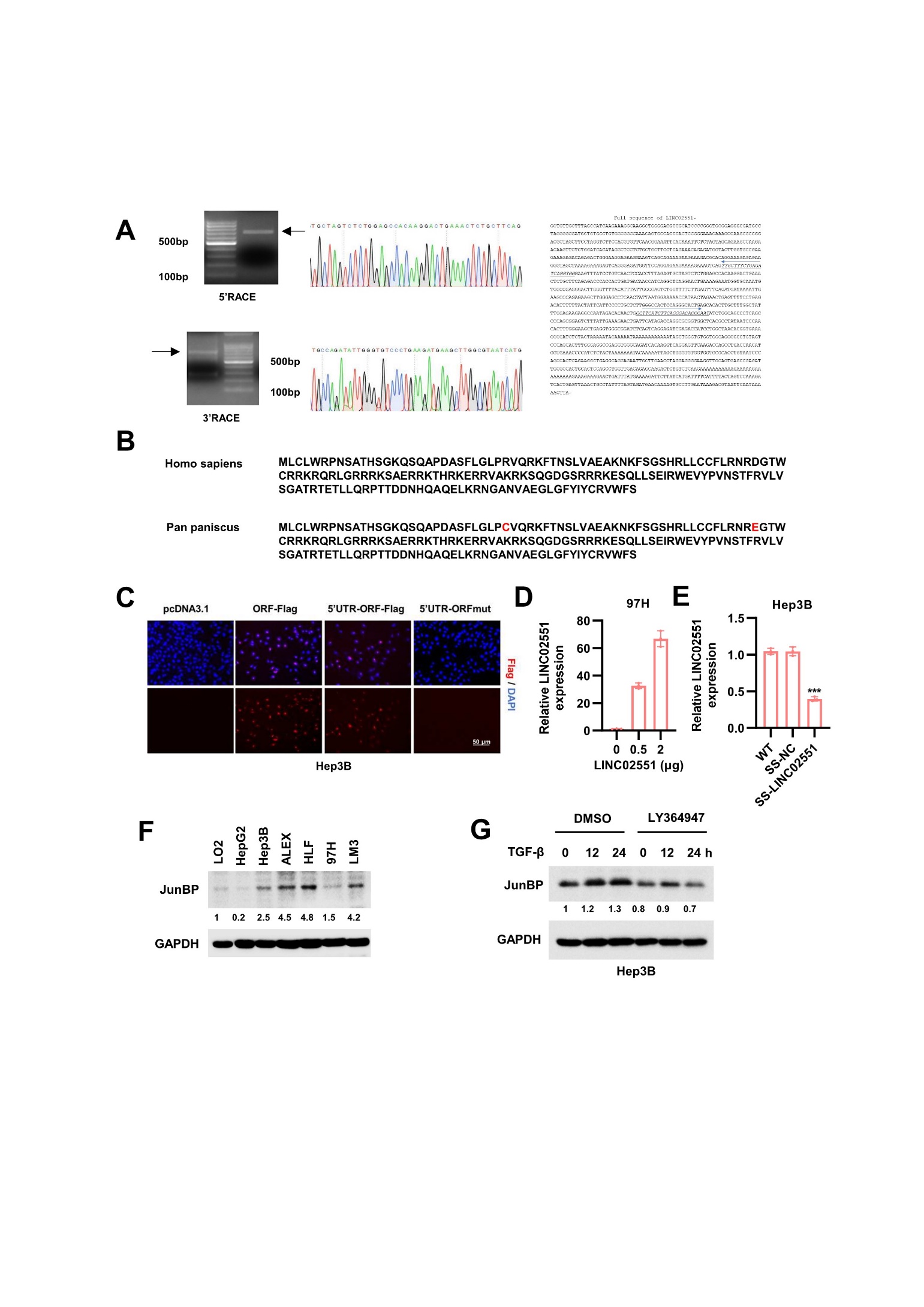


**Figure S2. The confirmation of JunBP. A)** 3’ and 5’ RACE result confirmed the sequence of LINC02551. **B)** The encoded peptide was blasted in NCBI and only expressed in human and Pan paniscus. **C)** The IF assays were performed in Hep3B cells transfected with indicated plasmids for 48 hours. **D)** qRT-PCR analysis of LINC02551 in 97H cells transfected with LINC02551 at indicated concentration. **E)** qRT-PCR analysis of LINC02551 in Hep3B transfected with LINC02551 Smart Silence. **F)** JunBP expression in different HCC cell lines. **G)** The expression of JunBP in Hep3B cells treated with TGF-β and LY364947 for indicated times. (mean ± SD, **P* < 0.05; ***P* < 0.01, ****P* < 0.001 and ns, *P* > 0.05).


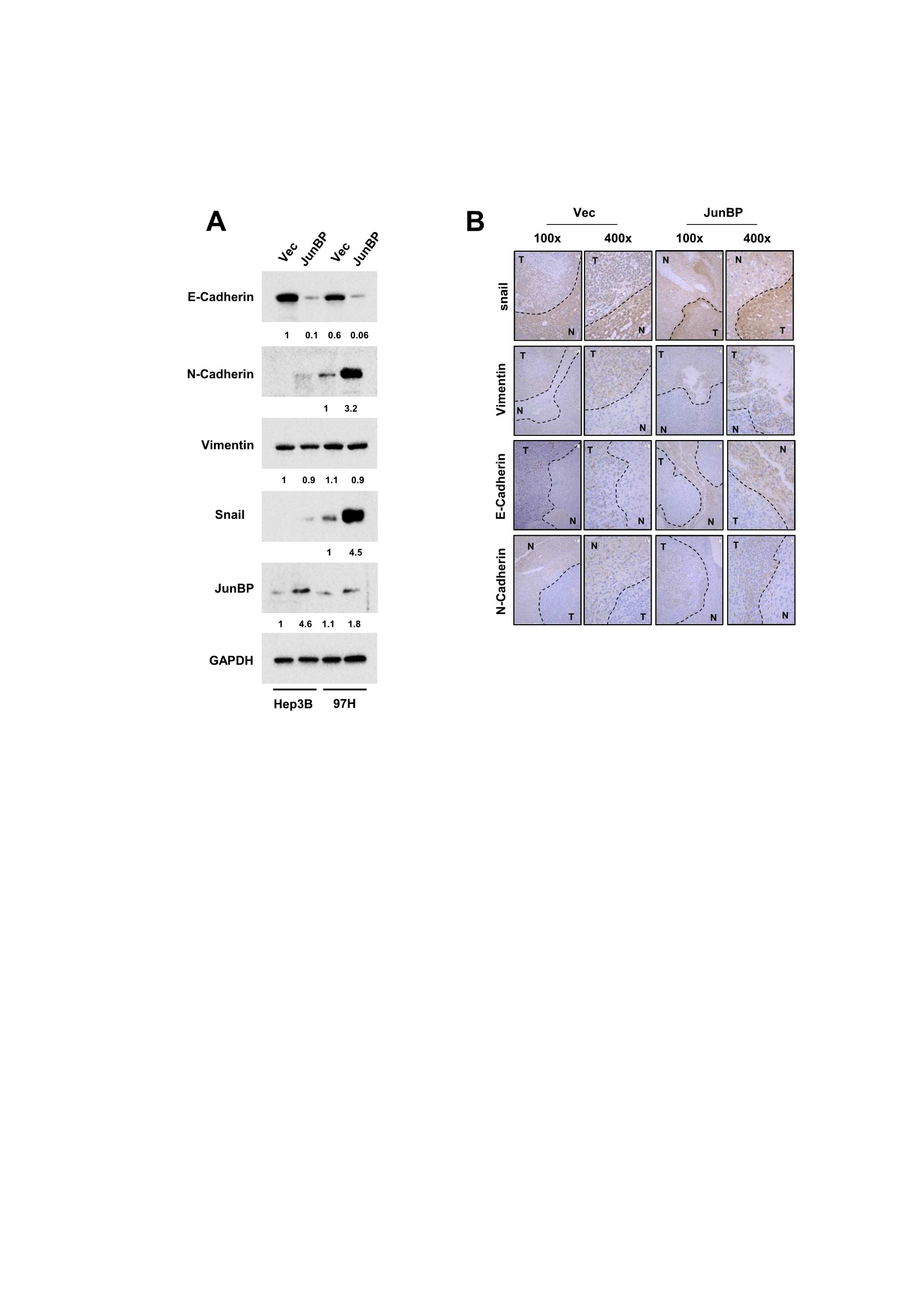


**Figure S3. The change of EMT markers when JunBP is upregulated. A)** WB analysis of EMT markers in Hep3B and 97h cells with JunBP overexpression. **B)** The IF staining of EMT markers of mice livers with 100🞨 magnification and 400🞨 magnification (T, tumor tissues; N, non-tumor tissues).


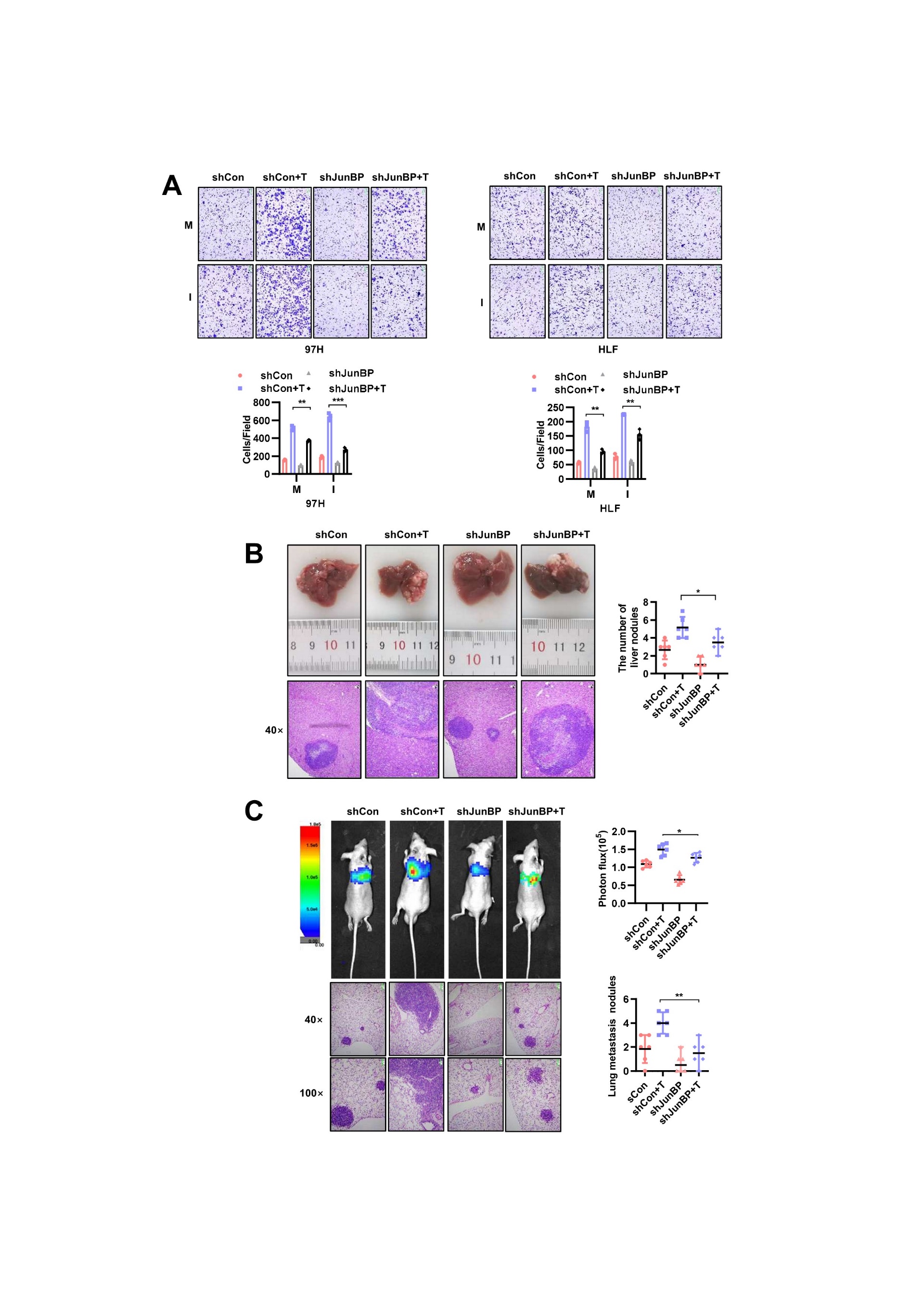


**Figure S4. The functional effects of JunBP knockdown upon TGF-β stimulation. A)** The migration (M) and invasion (I) results of JunBP knockdown with TGF-β stimulation in 97H and HLF cells. The lower panel is their statistic results. **B)** The livers separated from the mice of the indicated groups. The H&E staining results were presented below with 40🞨 magnification. The numbers of the liver tumor nodules from each group were calculated in the right panel. **C)** The mice were injected with luciferase substrate. The representative images from each group were presented in the upper panel. The H&E staining from the lungs were photographed with 40🞨 magnification and 100🞨 magnification. The statistical results were presented in the right panel. (mean ± SD, **P* < 0.05; ***P* < 0.01, ****P* < 0.001 and ns, *P* > 0.05).


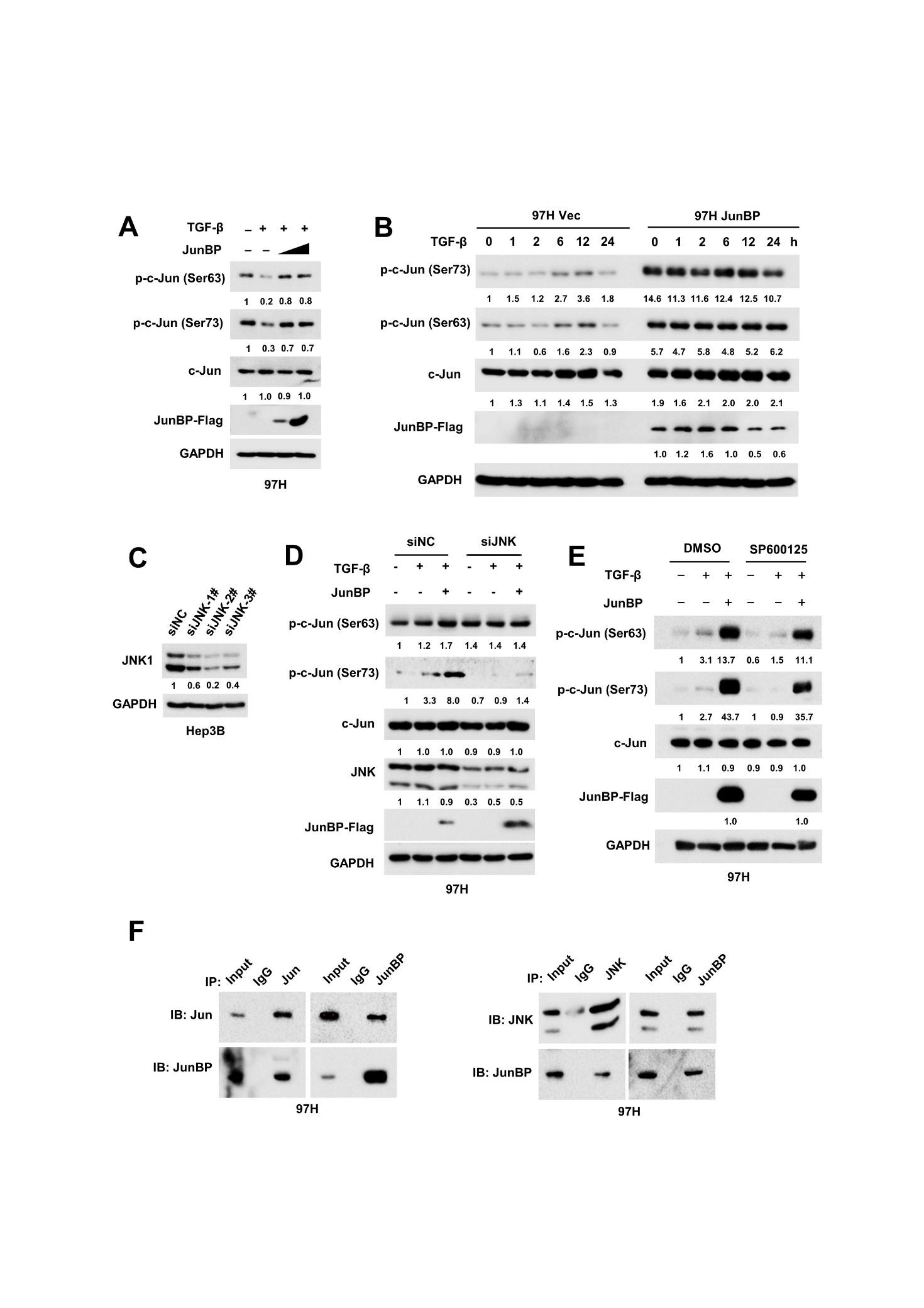


**Figure S5. The activation of c-Jun induced by JunBP. A)** WB analysis of 97H cells transfected with different concentrations of Flag-JunBP and treated with TGF-β. **B)** In 97H-Vector and 97H-JunBP-Flag cells, TGF-β were added for the indicated times and then indicated proteins were determined through WB. **C)** The confirmation of the knockdown efficiency of three sequences of siJNK in 97H cells through WB and siJNK-2# was chose for the further study. **D)** 97H cells were transfected with siJNK and Flag-JunBP for 48 hours and then treated with TGF-β for 30 min. **E)** 97H cells were transfected with JunBP and then treated with TGF-β and SP600125. **F)** The endogenous IP experiments were conducted in wild type 97H cells with anti-c-Jun, anti-JunBP, and anti-JNK.


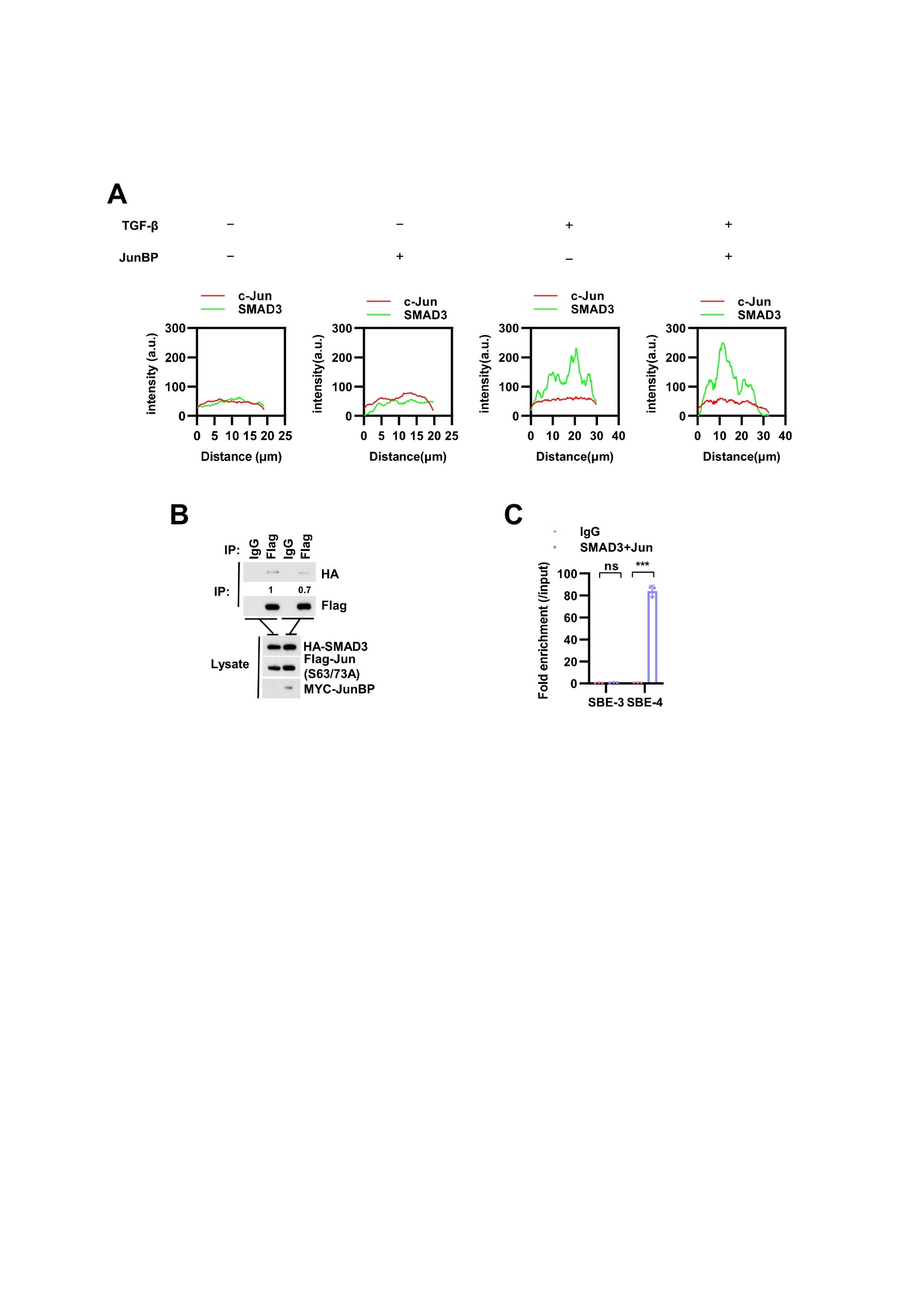


**Figure S6. The role of JunBP in the combination of SMAD3 and c-Jun. A)** The fluorescence intensities of figure 6A in the nucleus were quantified. **B)** When transfected with additional JunBP, the binding affinity between SMAD3 and c-Jun (S63/73A) is not increased. **C)** Re-ChIP assays using anti-c-Jun and anti-SMAD3 followed by rt-PCR in 97H cells.


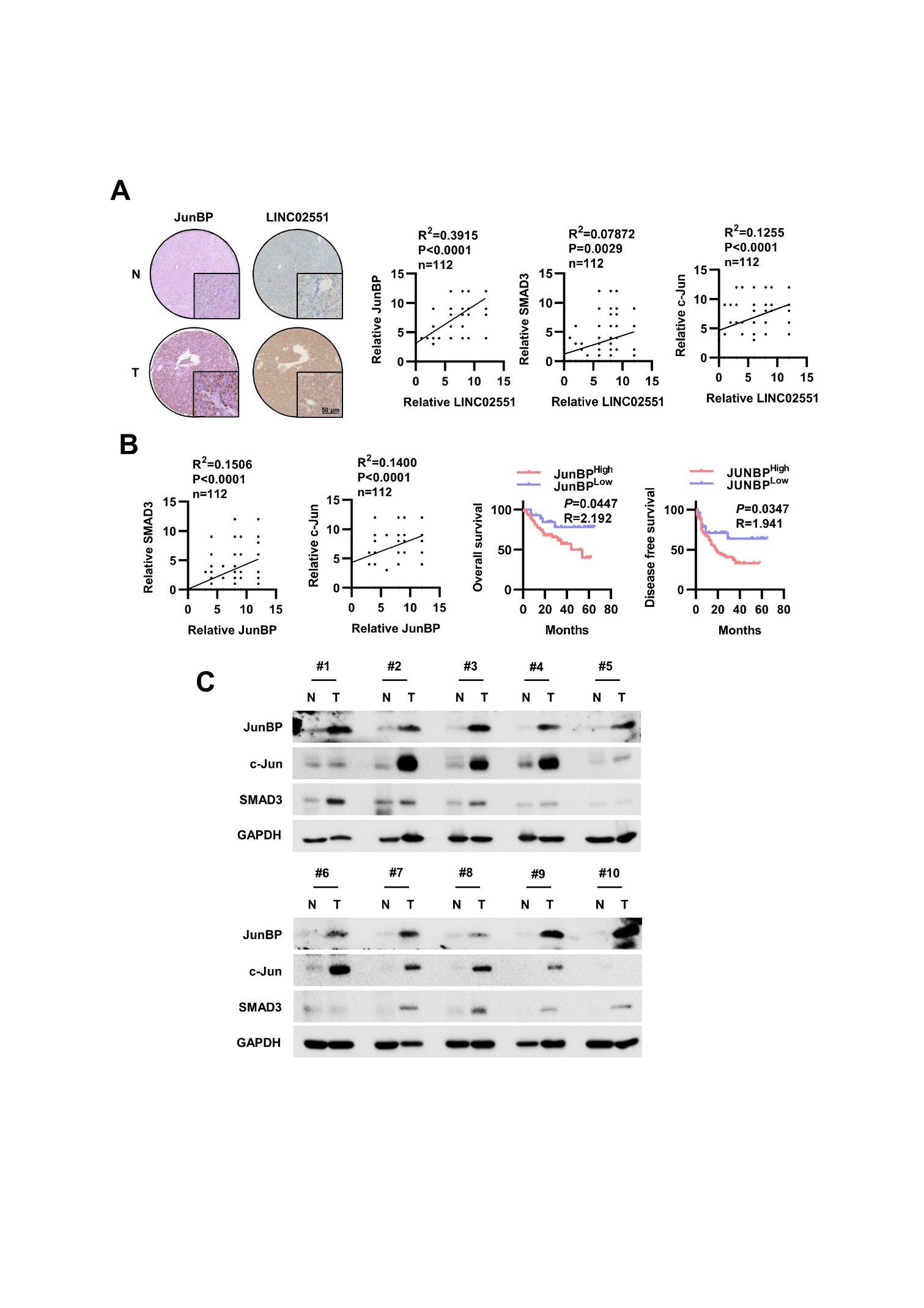


**Figure S7. The expression correlation among LINC02551, JunBP, c-Jun, and SMAD3 in HCC samples. A)** The representative IHC staining of paired tumor tissue (T) and non-tumor tissue (N) with anti-JunBP and ISH staining with digoxigenin-labeled LINC02551 in the same slide. And the expression correlation among LINC02551/JunBP, LINC02551/SMAD3, and LINC02551/c-Jun. **B)** The expression correlation between JunBP/SMAD3 and JunBP/c-Jun. And the OS and DFS of HCC patients with relatively high JunBP expression and relatively low JunBP expression. **C)** The representative WB images of the expression of JunBP, c-Jun, and SMAD3 in HCC patients.

**Table S1. Gene microarray results of TGF-β(5ng/ml) stimulation for 12 hours in HLF cells.**

| Vec_12h | Vec_0h | gene_name | log2FoldChange | pvalue | padj |
| --- | --- | --- | --- | --- | --- |
| 58.48018 | 10.4043 | CCDC144NL-AS1 | 2.48917 | 3.04E-10 | 1.00E-08 |
| **75.05621** | **13.75559** | **LINC02551** | **2.445271** | **6.45E-13** | **2.89E-11** |
| 97.82049 | 26.25094 | AP000695.1 | 1.897629 | 3.50E-12 | 1.45E-10 |
| 45.70766 | 13.14531 | MIAT | 1.802012 | 6.15E-06 | 1.00E-04 |
| 203.4142 | 61.09919 | NKILA | 1.734542 | 6.09E-18 | 4.15E-16 |
| 38.86419 | 12.084 | AL121759.2 | 1.682992 | 3.98E-05 | 0.00054 |
| 60.7546 | 20.47241 | AP000695.2 | 1.566125 | 2.79E-06 | 4.87E-05 |
| 55.77437 | 19.85402 | ADAMTS7P3 | 1.491321 | 5.91E-06 | 9.63E-05 |
| 34.20895 | 13.80339 | TMEM92-AS1 | 1.309733 | 0.001531 | 0.012683 |
| 121.5464 | 49.03772 | GASAL1 | 1.307741 | 5.61E-09 | 1.58E-07 |
| 87.86574 | 35.90968 | AL360004.1 | 1.290357 | 6.19E-07 | 1.24E-05 |
| 46.90274 | 19.4415 | HLA-H | 1.271167 | 0.003094 | 0.022974 |
| 80.49385 | 35.32999 | AC104825.1 | 1.186828 | 1.39E-05 | 0.000206 |
| 113.5262 | 50.09711 | AC022733.2 | 1.180656 | 1.50E-07 | 3.44E-06 |
| 34.89611 | 15.45863 | AL450043.1 | 1.173398 | 0.003886 | 0.027676 |
| 99.13505 | 44.42007 | CSPG4P13 | 1.157751 | 3.72E-06 | 6.35E-05 |
| 38.54676 | 17.51421 | AP000941.1 | 1.138062 | 0.003048 | 0.022718 |
| 90.70427 | 41.69434 | AC131009.2 | 1.119688 | 7.75E-06 | 0.000122 |
| 36.86599 | 17.09672 | AC131009.4 | 1.109203 | 0.006163 | 0.039981 |
| 115.8396 | 53.77023 | AC005077.4 | 1.108275 | 2.10E-06 | 3.78E-05 |
| 113.362 | 52.71603 | AC013652.1 | 1.103512 | 4.13E-07 | 8.57E-06 |
| 41.80333 | 19.8326 | AC004585.1 | 1.07474 | 0.003256 | 0.023961 |
| 71.50091 | 34.27375 | AC068282.1 | 1.059086 | 0.000107 | 0.001301 |
| 39.6969 | 19.16134 | KCNK15-AS1 | 1.051206 | 0.003575 | 0.025815 |
| 93.90093 | 45.67832 | LINC00322 | 1.039061 | 9.19E-06 | 0.000144 |
| 56.08617 | 27.54473 | AL157937.1 | 1.025826 | 0.000839 | 0.007577 |
| 182.3177 | 90.38164 | AC145098.2 | 1.0111 | 5.38E-09 | 1.53E-07 |
| 115.8117 | 59.44499 | AL139393.2 | 0.961886 | 6.73E-06 | 0.000108 |
| 66.01848 | 33.9214 | AC136632.1 | 0.960056 | 0.001588 | 0.013067 |
| 220.0687 | 114.8908 | AMZ2P1 | 0.937751 | 9.93E-10 | 3.08E-08 |
| 103.3643 | 55.14736 | AP001372.2 | 0.906848 | 4.46E-05 | 0.000596 |
| 114.6654 | 62.13932 | CASC15 | 0.883051 | 2.56E-05 | 0.000359 |
| 69.38691 | 38.36243 | SUCLG2-AS1 | 0.857024 | 0.001583 | 0.013045 |
| 334.086 | 190.5371 | AP000924.1 | 0.810587 | 2.87E-11 | 1.08E-09 |
| 51.53937 | 29.58291 | AL008729.1 | 0.801038 | 0.008157 | 0.049849 |
| 153.3165 | 89.45158 | AC004817.3 | 0.777626 | 1.34E-05 | 0.0002 |
| 106.3596 | 62.17399 | AL662907.2 | 0.774509 | 0.000337 | 0.003527 |
| 124.756 | 73.29939 | AC108062.1 | 0.768478 | 0.000198 | 0.002241 |
| 211.0516 | 125.3563 | AC010754.1 | 0.751564 | 7.53E-07 | 1.49E-05 |
| 158.6738 | 94.94692 | NUS1P1 | 0.74231 | 9.30E-05 | 0.001151 |
| 86.02664 | 51.83062 | AC126755.1 | 0.730097 | 0.002832 | 0.021349 |
| 135.3493 | 81.97402 | AC122710.2 | 0.723928 | 0.000121 | 0.001453 |
| 92.01485 | 56.82093 | SMG1P5 | 0.696816 | 0.001931 | 0.015507 |
| 130.3728 | 83.94713 | AP000759.1 | 0.636439 | 0.001469 | 0.012263 |
| 107.5396 | 69.27567 | LINC00863 | 0.635384 | 0.001908 | 0.015348 |
| 191.7832 | 124.7572 | CACTIN-AS1 | 0.619096 | 0.000178 | 0.002036 |
| 280.3696 | 183.3005 | AC018629.1 | 0.613471 | 4.69E-06 | 7.83E-05 |
| 280.4924 | 185.5275 | AL358334.2 | 0.597547 | 9.43E-06 | 0.000147 |
| 186.4536 | 124.2929 | LINC01137 | 0.585548 | 0.000266 | 0.002888 |
| 1553.565 | 1035.629 | AC020916.1 | 0.585374 | 4.67E-22 | 4.23E-20 |
| 800.4551 | 542.6613 | LINC02081 | 0.561415 | 9.41E-13 | 4.17E-11 |
| 0.985935 | 0.67027 | LINC01353 | 0.554434 | 0.795844 | 1 |
| 198.8017 | 137.7899 | FAM13A-AS1 | 0.530046 | 0.003545 | 0.02565 |
| 443.3195 | 311.0026 | AC021054.1 | 0.511465 | 3.32E-07 | 6.98E-06 |
| 259.6854 | 182.5559 | AC144652.1 | 0.508557 | 9.78E-05 | 0.001205 |
| 163.0826 | 115.3237 | AC009948.1 | 0.499365 | 0.002354 | 0.018336 |
| 318.608 | 227.0087 | AL109615.3 | 0.489604 | 3.78E-05 | 0.000516 |
| 346.8472 | 248.9265 | GAS6-AS1 | 0.479185 | 6.95E-05 | 0.000886 |
| 188.4594 | 135.2356 | AL590560.1 | 0.478474 | 0.003204 | 0.023659 |
| 179.4252 | 132.4905 | LINC01106 | 0.437279 | 0.004785 | 0.032889 |
| 201.5976 | 148.9871 | LINC01537 | 0.435802 | 0.003287 | 0.024114 |
| 303.6869 | 225.7797 | FRG1CP | 0.426523 | 0.000778 | 0.007132 |
| 4329.063 | 3226.123 | LINC00963 | 0.423992 | 1.51E-26 | 1.92E-24 |
| 345.4133 | 261.0047 | AC245041.1 | 0.403448 | 0.000618 | 0.005892 |
| 285.7844 | 218.9879 | AC013444.1 | 0.385621 | 0.003744 | 0.026769 |
| 511.1582 | 408.3169 | TMSB4XP8 | 0.324661 | 0.000913 | 0.008151 |
| 597.3042 | 477.3024 | LINC00942 | 0.323971 | 0.000161 | 0.001869 |
| 605.6476 | 484.9182 | AC016717.2 | 0.320656 | 0.000139 | 0.001636 |
| 759.7497 | 622.561 | MT-TY | 0.28822 | 0.000533 | 0.005225 |
| 563.3065 | 464.6336 | AC074117.1 | 0.277996 | 0.001437 | 0.012019 |
| 1111.247 | 929.4725 | RPSAP58 | 0.2578 | 0.000223 | 0.002477 |
| 585.3981 | 495.9911 | SH3BP5-AS1 | 0.240205 | 0.008112 | 0.049664 |
| 3373.461 | 2932.571 | MT-TC | 0.201833 | 2.56E-07 | 5.51E-06 |
| 5745.536 | 5225.551 | FGD5-AS1 | 0.136857 | 5.65E-05 | 0.000735 |
| 155971.7 | 142196.5 | MT-RNR2 | 0.133383 | 3.11E-05 | 0.000432 |
| 3487.215 | 3765.834 | GAS5 | -0.11098 | 0.004905 | 0.033613 |
| 2177.915 | 2390.722 | RPL6P27 | -0.1347 | 0.005301 | 0.035601 |
| 1154.847 | 1311.937 | RPL13AP5 | -0.18423 | 0.001392 | 0.011706 |
| 8621.881 | 9809.902 | RPS2P5 | -0.18644 | 6.91E-06 | 0.000111 |
| 1483.378 | 1695.875 | SNHG5 | -0.19355 | 0.000756 | 0.006965 |
| 825.3091 | 948.8789 | LINC01578 | -0.20106 | 0.004342 | 0.030292 |
| 853.612 | 982.4882 | RPL13P12 | -0.20333 | 0.006403 | 0.041242 |
| 967.1906 | 1116.525 | RPL3P4 | -0.20764 | 0.004472 | 0.031052 |
| 946.5944 | 1094.398 | DANCR | -0.20939 | 0.000885 | 0.007943 |
| 603.9394 | 701.8747 | EPB41L4A-AS1 | -0.21722 | 0.005767 | 0.037956 |
| 643.5706 | 757.8666 | SNHG17 | -0.23542 | 0.005541 | 0.03674 |
| 760.811 | 902.5849 | SNHG4 | -0.24658 | 0.0003 | 0.003182 |
| 5423.984 | 6483.814 | CASC19 | -0.25735 | 0.000593 | 0.005696 |
| 561.2603 | 688.5079 | NPM1P27 | -0.29545 | 0.000218 | 0.002435 |
| 352.7649 | 438.7569 | SNHG12 | -0.31454 | 0.005254 | 0.035355 |
| 489.7846 | 618.7863 | PURPL | -0.33642 | 0.001318 | 0.011218 |
| 566.7889 | 724.1795 | SNHG7 | -0.35294 | 6.29E-06 | 0.000102 |
| 233.9259 | 302.7044 | AC016739.1 | -0.3724 | 0.005461 | 0.036334 |
| 193.8014 | 259.3812 | AL365181.3 | -0.42005 | 0.001278 | 0.010958 |
| 130.4851 | 177.1004 | SNHG19 | -0.44148 | 0.00423 | 0.029633 |
| 796.7595 | 1112.19 | AL161431.1 | -0.48065 | 2.68E-11 | 1.02E-09 |
| 110.7196 | 154.6397 | BASP1-AS1 | -0.48069 | 0.005118 | 0.034648 |
| 121.2716 | 171.899 | AC241952.1 | -0.50072 | 0.003398 | 0.024706 |
| 249.7718 | 357.9485 | AC010343.1 | -0.5194 | 4.72E-05 | 0.000626 |
| 96.66835 | 138.74 | FTH1P2 | -0.52245 | 0.003663 | 0.026314 |
| 216.717 | 314.4948 | AL136164.4 | -0.53669 | 3.14E-05 | 0.000435 |
| 163.5252 | 237.8739 | AC007969.1 | -0.54209 | 0.000165 | 0.001907 |
| 348.0857 | 515.2881 | BX679664.3 | -0.56705 | 3.65E-06 | 6.26E-05 |
| 70.4457 | 106.2923 | PRKAG2-AS1 | -0.59203 | 0.005779 | 0.038019 |
| 79.42929 | 120.2019 | RAET1E-AS1 | -0.59801 | 0.007418 | 0.046215 |
| 73.12718 | 112.5916 | EWSAT1 | -0.62199 | 0.002374 | 0.018464 |
| 51.51509 | 79.6596 | PRRT3-AS1 | -0.62853 | 0.008036 | 0.049264 |
| 133.7846 | 206.9899 | AC009275.1 | -0.63054 | 3.41E-05 | 0.000468 |
| 168.9307 | 262.1077 | LINC00472 | -0.63224 | 3.51E-06 | 6.02E-05 |
| 58.06178 | 90.65071 | RPL26P19 | -0.64279 | 0.00573 | 0.037829 |
| 470.6588 | 756.2055 | LINC02009 | -0.68483 | 1.34E-17 | 8.98E-16 |
| 35.78307 | 61.54229 | AL357033.4 | -0.78204 | 0.004576 | 0.031665 |
| 33.13897 | 58.14011 | LDHAP4 | -0.81071 | 0.004175 | 0.029294 |
| 303.6734 | 533.6077 | H19 | -0.81275 | 6.20E-11 | 2.26E-09 |
| 28.71353 | 51.09014 | C1QTNF1-AS1 | -0.83467 | 0.007599 | 0.04708 |
| 156.9596 | 296.5432 | DUBR | -0.91655 | 5.55E-12 | 2.28E-10 |
| 9.560578 | 38.6406 | AC139749.1 | -2.01863 | 4.01E-06 | 6.82E-05 |

**Table S2. Proteins bind with JunBP identified by IP-MS.**

| Gene Name | #Unique Peptides | #Protein Groups | # AAs | MW [kDa] |
| --- | --- | --- | --- | --- |
| THOC2 | 15 | 1 | 1593 | 182.659 |
| RPL13A | 13 | 1 | 203 | 23.562 |
| RPS9 | 12 | 1 | 194 | 22.578 |
| EXOSC10 | 11 | 1 | 885 | 100.768 |
| PRKDC | 10 | 1 | 4128 | 468.788 |
| GTPBP4 | 10 | 1 | 634 | 73.918 |
| RBMX | 10 | 1 | 391 | 42.306 |
| DHX37 | 10 | 1 | 1157 | 129.464 |
| AQR | 10 | 1 | 1485 | 171.186 |
| PBRM1 | 10 | 1 | 1689 | 192.825 |
| IRS4 | 10 | 1 | 1257 | 133.685 |
| RPL21 | 9 | 1 | 160 | 18.553 |
| RBM34 | 8 | 1 | 430 | 48.535 |
| PES1 | 8 | 1 | 588 | 67.96 |
| NKRF | 8 | 1 | 690 | 77.624 |
| NOC3L | 7 | 1 | 800 | 92.49 |
| EIF2S3 | 7 | 1 | 472 | 51.077 |
| G3BP2 | 7 | 1 | 482 | 54.088 |
| SCAF4 | 7 | 1 | 1147 | 125.79 |
| PUM3 | 7 | 1 | 648 | 73.538 |
| RPL36 | 7 | 1 | 105 | 12.246 |
| MAP4K4 | 7 | 1 | 1239 | 142.013 |
| RPL34 | 7 | 1 | 117 | 13.284 |
| TRA2A | 6 | 1 | 282 | 32.669 |
| YBX1 | 6 | 1 | 324 | 35.903 |
| **JUN** | 6 | 1 | 331 | 35.653 |
| SFPQ | 6 | 1 | 707 | 76.102 |
| HNRNPK | 6 | 1 | 463 | 50.944 |
| ADAR | 6 | 1 | 1226 | 135.981 |
| TSR1 | 6 | 1 | 804 | 91.752 |
| FMR1 | 6 | 1 | 632 | 71.131 |
| SCAF1 | 6 | 1 | 1312 | 139.186 |
| AP2A1 | 6 | 1 | 977 | 107.478 |
| RPL36A | 6 | 1 | 106 | 12.433 |
| RBM15 | 6 | 1 | 977 | 107.124 |
| ATXN2L | 6 | 1 | 1075 | 113.304 |
| FXR1 | 6 | 1 | 621 | 69.678 |
| PRMT5 | 6 | 1 | 637 | 72.638 |
| WDR12 | 6 | 1 | 423 | 47.678 |
| PWP2 | 6 | 1 | 919 | 102.387 |
| GTF3C1 | 6 | 1 | 2109 | 238.725 |
| MRTO4 | 6 | 1 | 239 | 27.543 |
| SMARCA5 | 6 | 1 | 1052 | 121.828 |
| DDX27 | 6 | 1 | 796 | 89.779 |
| U2AF1 | 5 | 1 | 240 | 27.854 |
| SRSF10 | 5 | 1 | 262 | 31.282 |
| RPS27A | 5 | 1 | 156 | 17.953 |
| CDK12 | 5 | 1 | 1490 | 164.054 |
| RNPS1 | 5 | 1 | 305 | 34.188 |
| RPL35 | 5 | 1 | 123 | 14.543 |
| PRPF40A | 5 | 1 | 957 | 108.737 |
| GAPDH | 5 | 1 | 335 | 36.03 |
| H1-3 | 5 | 1 | 221 | 22.336 |
| USP39 | 5 | 1 | 565 | 65.34 |
| PHF6 | 5 | 1 | 365 | 41.264 |
| FXR2 | 5 | 1 | 673 | 74.178 |
| RBM28 | 5 | 1 | 759 | 85.685 |
| DDX31 | 5 | 1 | 851 | 94.029 |
| SAFB | 5 | 1 | 915 | 102.58 |
| RBBP6 | 5 | 1 | 1792 | 201.442 |
| RPL22 | 5 | 1 | 128 | 14.778 |
| NOLC1 | 5 | 1 | 699 | 73.56 |
| SNRPB | 5 | 1 | 240 | 24.594 |
| PELP1 | 5 | 1 | 1130 | 119.624 |
| SF3B2 | 5 | 1 | 895 | 100.165 |
| MACROH2A1 | 5 | 1 | 372 | 39.592 |
| KRT9 | 5 | 1 | 623 | 62.027 |
| USP10 | 5 | 1 | 798 | 87.08 |
| CLTC | 5 | 1 | 1675 | 191.493 |
| RFC1 | 5 | 1 | 1148 | 128.175 |
| ZCCHC3 | 5 | 1 | 403 | 43.52 |
| GEMIN4 | 5 | 1 | 1058 | 119.96 |
| RPS10 | 5 | 1 | 165 | 18.886 |
| RPS16 | 5 | 1 | 146 | 16.435 |
| CPSF1 | 5 | 1 | 1443 | 160.782 |
| HNRNPF | 4 | 1 | 415 | 45.643 |
| CPSF6 | 4 | 1 | 551 | 59.173 |
| SLC25A5 | 4 | 1 | 298 | 32.831 |
| SRSF2 | 4 | 1 | 221 | 25.461 |
| RPS26 | 4 | 1 | 115 | 13.007 |
| SREK1 | 4 | 1 | 508 | 59.345 |
| NONO | 4 | 1 | 471 | 54.197 |
| LYAR | 4 | 1 | 379 | 43.588 |
| NEMF | 4 | 1 | 1076 | 122.878 |
| EIF4A3 | 4 | 1 | 411 | 46.841 |
| RRP9 | 4 | 1 | 475 | 51.809 |
| COP1 | 4 | 1 | 731 | 80.424 |
| SRPK2 | 4 | 1 | 688 | 77.478 |
| PPP1CA | 4 | 1 | 330 | 37.488 |
| NIFK | 4 | 1 | 293 | 34.201 |
| PRPF31 | 4 | 1 | 499 | 55.421 |
| PRPF38A | 4 | 1 | 312 | 37.453 |
| TJP2 | 4 | 1 | 1190 | 133.876 |
| DSP | 4 | 1 | 2871 | 331.569 |
| PRPF3 | 4 | 1 | 683 | 77.481 |
| SLC25A3 | 4 | 1 | 362 | 40.069 |
| RPL19 | 4 | 1 | 196 | 23.451 |
| ZNF326 | 4 | 1 | 582 | 65.613 |
| NOM1 | 4 | 1 | 860 | 96.198 |
| GNL2 | 4 | 1 | 731 | 83.603 |
| RSRC2 | 4 | 1 | 434 | 50.529 |
| SRSF9 | 4 | 1 | 221 | 25.526 |
| RPL38 | 4 | 1 | 70 | 8.213 |
| RPL7L1 | 4 | 1 | 255 | 29.651 |
| SNRPD2 | 4 | 1 | 118 | 13.518 |
| PPIG | 4 | 1 | 754 | 88.564 |
| UTP15 | 4 | 1 | 518 | 58.379 |
| SVIL | 4 | 1 | 2214 | 247.593 |
| GAR1 | 4 | 1 | 217 | 22.334 |
| IARS1 | 4 | 1 | 1262 | 144.406 |
| DDX10 | 4 | 1 | 875 | 100.825 |
| ZFC3H1 | 4 | 1 | 1989 | 226.214 |
| SSB | 4 | 1 | 408 | 46.808 |
| TNRC6A | 4 | 1 | 1962 | 210.169 |
| ZFP41 | 4 | 1 | 198 | 22.791 |
| XRN1 | 4 | 1 | 1706 | 193.985 |
| TAF15 | 3 | 1 | 592 | 61.793 |
| SNRPD1 | 3 | 1 | 119 | 13.273 |
| MYH14 | 3 | 1 | 1995 | 227.732 |
| NACA | 3 | 1 | 2078 | 205.295 |
| RPL37A | 3 | 1 | 92 | 10.268 |
| KRI1 | 3 | 1 | 703 | 82.548 |
| PCBP1 | 3 | 1 | 356 | 37.474 |
| RPS12 | 3 | 1 | 132 | 14.505 |
| NOL9 | 3 | 1 | 702 | 79.272 |
| LARP7 | 3 | 1 | 582 | 66.857 |
| DAZAP1 | 3 | 1 | 407 | 43.356 |
| LIMA1 | 3 | 1 | 759 | 85.173 |
| EXOSC7 | 3 | 1 | 291 | 31.801 |
| RPS13 | 3 | 1 | 151 | 17.212 |
